# Two extended haplotype blocks are associated with adaptation to high altitude habitats in East African honey bees

**DOI:** 10.1371/journal.pgen.1006792

**Published:** 2017-05-25

**Authors:** Andreas Wallberg, Caspar Schöning, Matthew T. Webster, Martin Hasselmann

**Affiliations:** 1 Department of Medical Biochemistry and Microbiology, Science for Life Laboratory, Uppsala University, Uppsala, Sweden; 2 Institute for Bee Research, Hohen Neuendorf, Germany; 3 Department of Livestock Population Genomics, Institute of Animal Science, University of Hohenheim, Stuttgart, Germany; Queen Mary University of London, UNITED KINGDOM

## Abstract

Understanding the genetic basis of adaption is a central task in biology. Populations of the honey bee *Apis mellifera* that inhabit the mountain forests of East Africa differ in behavior and morphology from those inhabiting the surrounding lowland savannahs, which likely reflects adaptation to these habitats. We performed whole genome sequencing on 39 samples of highland and lowland bees from two pairs of populations to determine their evolutionary affinities and identify the genetic basis of these putative adaptations. We find that in general, levels of genetic differentiation between highland and lowland populations are very low, consistent with them being a single panmictic population. However, we identify two loci on chromosomes 7 and 9, each several hundred kilobases in length, which exhibit near fixation for different haplotypes between highland and lowland populations. The highland haplotypes at these loci are extremely rare in samples from the rest of the world. Patterns of segregation of genetic variants suggest that recombination between haplotypes at each locus is suppressed, indicating that they comprise independent structural variants. The haplotype on chromosome 7 harbors nearly all octopamine receptor genes in the honey bee genome. These have a role in learning and foraging behavior in honey bees and are strong candidates for adaptation to highland habitats. Molecular analysis of a putative breakpoint indicates that it may disrupt the coding sequence of one of these genes. Divergence between the highland and lowland haplotypes at both loci is extremely high suggesting that they are ancient balanced polymorphisms that greatly predate divergence between the extant honey bee subspecies.

## Author summary

Identifying the genes and genetic changes responsible for environmental adaptation is an important step towards understanding how species evolve. The honey bee *Apis mellifera* has adapted to a variety of habitats across its worldwide geographical distribution. Here we aim to identify the genetic basis of adaptation in honey bees living at high altitudes in the mountains of East Africa, which differ in appearance and behavior from their lowland relatives. We compare whole genome sequences from highland and lowland populations and find that, although in general they are extremely similar, there are two specific chromosomal regions (representing 1.4% of the genome) where they are strongly differentiated. These regions appear to represent structural rearrangements that are strongly correlated with altitude and contain many genes. One of these genomic regions harbors a set of octopamine receptor genes, which we hypothesize regulate differences in learning and foraging behavior between highland and lowland bees. The extremely high divergence between highland and lowland genetic variants in these regions indicates that they have an ancient origin and were likely to have been involved in environmental adaptation even before honey bees came to inhabit their current range.

## Introduction

Genetic adaptation to different environmental conditions is a key process in evolution and speciation. However, identifying the genetic variants involved in adaptation and the underlying regulatory networks and biological mechanisms by which they impact fitness is challenging. There are relatively few instances where the genetic basis of environmental adaptation is well understood [1]. Some examples where genetic variation has been linked to locally adaptive phenotypic differences are the pigmentation differences in the rock pocket mouse driven by variation in at least one melanocortin receptor [2], the industrial melanism of the peppered moth driven by a transposal element in the *cortex* gene [3,4] and the adaptive evolution of populations of sticklebacks [5], including the pelvic reduction driven by recurrent deletion of a tissue specific enhancer [6].

Studies of highland populations have proven informative for understanding the genetic basis of adaptation [7–11]. First, they inhabit different environments in close proximity to lowland populations. Genetic exchange or recent ancestry between the highland and neighboring lowland populations is therefore likely to result in low differentiation in neutrally evolving markers between highland and lowland populations, making it easier to distinguish loci involved in local adaptation. Secondly, analysis of interconnected populations spanning different habitats affords the opportunity to determine how processes such as convergent evolution [12] or adaptation from standing variation [13] have contributed to their adaptations. For example, genomic analysis of genetic adaptation of human populations living at high altitudes on three continents have revealed that convergent evolution involving selection on variants in different genes related to adaptation to hypoxia are responsible for their adaptations [8,14–16]. Conversely, analysis of freshwater adaptation in sticklebacks has implicated that a suite of genetic variants are present in multiple geographically distant localities, implicating selection on standing variation [5,17].

Most genes demonstrated to be involved in adaptation have effects on morphology or physiology [1,18]. However, some studies have also identified putatively adaptive variation involved in differences in fitness related to behavior [19], such as genes that control variation in burrow architectures of *Peromyscus* mice [20]. Genome comparisons allow us to identify genes involved in local adaptations to different habitats where the phenotypic nature of these adaptations is not necessarily well characterized [18]. Many adaptations in social insects are likely to be behavioral [21]. In particular, honey bees have sophisticated cognitive abilities, which are needed to efficiently perform the diverse set of tasks necessary for optimal functioning of a colony. Furthermore, efficient foraging requires recognition of floral scents, location of flowers, association with a food reward and advertising food sources with a characteristic dance [22]. Optimal foraging strategies are likely to be variable between habitats and subject to selection [23].

The honey bee *Apis mellifera* has a large native range incorporating a wide variety of habitats. There is substantial variation in morphology, physiology and behavior across this range, which are likely to represent local adaptations [24,25]. The mountain regions of East Africa are highly complex in their topography with scattered high mountains, most of them of volcanic origin and comprising of three distinct vegetation belts: montane forest, subalpine heathlands and an alpine zone [26]. The average annual temperature of mountain rain forest habitats at 2600 m altitude is only 11.2°C, remarkably different from lowland savannah regions below 1500 m (20.8°C) [24].

The honey bees found in the mountain forests differ in phenotype compared to the bees of the savannah. They have been designated as a separate subspecies *A*. *m*. *monticola* Smith 1961 [27], whereas savannah bees have been assigned to *A*. *m*. *scutellata* Lepeletier de Saint Fargeau 1836 [24]. Mountain and savannah bees can be distinguished on the basis of morphometrics, although the status of *monticola* as a distinct subspecies from *scutellata* has been a matter of debate [28–32]. Bees from colonies identified as *monticola* tend to be darker in color, larger and less aggressive than *scutellata* savannah bees [24,27,33]. Measurement of mating frequencies indicates that levels of polyandry in *monticola* honey bees are significantly lower than in *scutellata* [34]. Descriptions of the behavior of *monticola* honey bees suggest that they can fly at lower temperatures than *scutellata* colonies, conserve honey stores during times of reduced nectar flow by reducing brood rearing and are less prone to abandon their nests by swarming or absconding [24,27,32,35,36]. It is therefore likely that *monticola* honey bees possess adaptations for life in cool mountain forests.

The population history of the mountain bees is debated. The mountain refugia hypothesis proposes that mountain bees have survived as small and reproductively isolated populations for thousands of generations [29,31]. Such isolation can be expected to result in distinctly different patterns of genetic diversity compared to the widespread lowland bees. The results of a study based on mtDNA data supported this scenario [31]. However, a separate study of mtDNA and microsatellites did not identify genetic differences between the *monticola* honey bees and surrounding lowland populations [28], which could suggest that the phenotypes observed in *monticola* honey bees represent phenotypic plasticity [28] or that they are determined genetically but have not led to reproductive isolation. A perennial hybridization of *monticola* with *scutellata* in a transitory zone of altitude has previously been reported [24,27].

**Fig 1:**
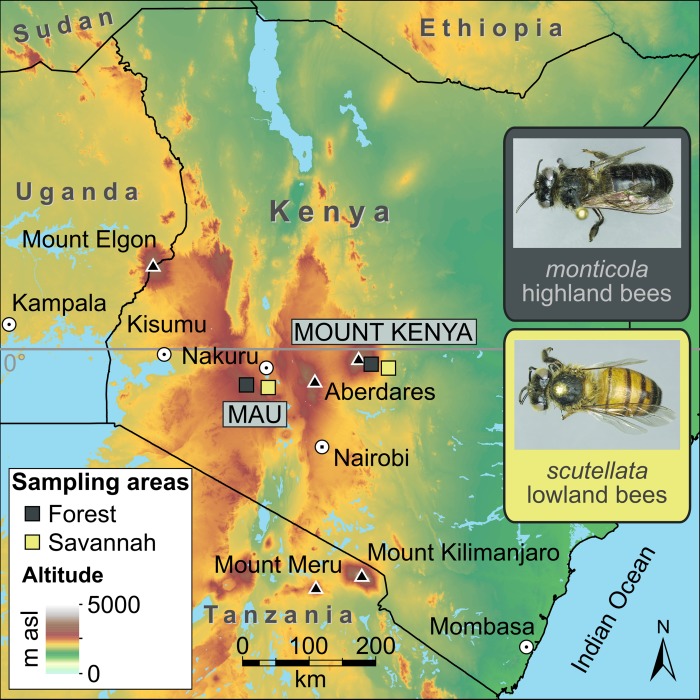
Map of Kenyan honey bee sample locations. The *monticola* bee is associated with the isolated highland forests and the *scutellata* bee occurs in the surrounding lowland savannahs. High mountain peaks where *monticola* has been found are indicated with black triangles. Mount Kenya and Mau boxes indicate sample locations (grey = *monticola*; yellow = *scutellata*). Mt Kenya Forest (average sample elevation 2,300 m above sea level; n = 10), Mt Kenya Savannah (1,100 m asl; n = 9), Mau Forest (2,900 m asl; n = 10) and Mau Savannah (1,900 m asl; n = 10).

In this study, we compare whole-genome sequences from 39 worker bees representing two Kenyan mountain areas that are approximately 100km apart: Mt Kenya and Mau (Fig 1). Each locality includes unmanaged bees from neighboring highland forest and lowland savannah environments that are separated by approximately 1000m in altitude. We aim to clarify the evolutionary origin of these populations and the genetic basis of their adaptation to high-altitude habitats.

## Results

### Population-scale sequencing of honey bees from highland and lowland habitats in Kenya

We mapped genome variation in the East African mountain honey bees (*A*. *m*. *monticola*) in order to infer the evolutionary history of the population and identify loci involved in adaptation to altitude. We sequenced 39 samples previously collected from four native feral populations [28]: Mt Kenya Forest (MKF, 2,300 m above sea level; n = 10), Mt Kenya Savannah (MKS, 1,100 m above sea level; n = 9), Mau Forest (MF, 2,900 m above sea level; n = 10) and Mau Savannah (MS, 1,900 m above sea level; n = 10) (Fig 1; S1A Table). The bees collected from the highland forest localities are referred to as *A*. *m*. *monticola* (hereafter *monticola*), whereas lowland savannah samples are referred to as *A*. *m*. *scutellata* (hereafter *scutellata*). Samples from the highland and lowland regions could be separated by morphometrics in a previous study [28]. We produced 490 million short-reads to generate a 463× dataset spanning the sixteen assembled nuclear chromosomes, unplaced contigs and mitochondrial genome (see Materials and methods). Some unplaced contigs and the mitochondrion in particular were sequenced at extremely high coverage, inflating the average coverage. The assembled nuclear genome was sequenced to 10.4× per sample (80% of the genome was covered by >5× per sample) and unless indicated otherwise, the results below refer to analyses of this data (S1A Table).

We next called single nucleotide polymorphisms (SNPs) across the 39 samples. 8.6 million biallelic SNPs were retained after filtering and imputation (Table 1). For some comparative analyses, we expanded the dataset to include previously published Kenyan honey bee genomes (n = 11; data from [37]) and a worldwide sample of honey bees (n = 98; data from [25]; S1B and S1C Table). This makes it possible to position the Mt Kenya and Mau samples among other honey bee populations and detect uniquely divergent regions in highland genomes. The expanded dataset was produced using the same methods and spanned 13.6 million SNPs. The genome sequence of the Eastern honey bee *A*. *cerana* [38] was aligned against the *A*. *mellifera* reference genome in order to further facilitate assessments of divergence and distinguish between ancestral and derived variants. The corresponding *A*. *cerana* sequence was present at 78% of the *A*. *mellifera* genome. Genome-wide divergence between the two species was estimated as 6.9%.

**Table 1:** Population genetic statistics.

Population samples				Diversity				Divergence (*F*_ST_)			
Locality	Environment	Label	n	SNPs (n)	*π (%/bp)*	*θ*_w_ (%/bp)	*N*_E_	MF	MS	MKF	MKS
Mau	Forest	MF	10	5,836,798	0.66	0.76	478,031	-	0.050	0.049	0.057
	Savannah	MS	10	5,931,732	0.67	0.78	488,189	0.050	-	0.065	0.046
Mt Kenya	Forest	MKF	10	5,731,149	0.65	0.75	469,852	0.049	0.065	-	0.067
	Savannah	MKS	9	5,550,554	0.66	0.75	470,880	0.057	0.046	0.067	-
	ALL		39	8,593,016	0.67	0.82	516,601				

Similar levels of genetic diversity and low differentiation between highland and lowland populations

The mountain refugia hypothesis suggests that *monticola* populations are small relicts that have been reproductively isolated from lowland *scutellata* bees [31]. This hypothesis makes predictions about genetic variation in highland bees compared to lowland bees. Assuming that small populations have comparatively low effective population sizes (*N*_E_), we can expect lower levels of neutral variation in *monticola* than in *scutellata* under equilibrium [39,40]. The number of SNPs detected within each of the four populations ranged between 5.5–5.9 million, corresponding to nearly identical estimates of nucleotide diversity (π = 0.65–0.67%/bp), the population mutation rate (*θ*_w_ = 0.75–0.78%/bp) and effective sizes of each population (*N*_E_ = 470×10^3^–488×10^3^) (Table 1). We do not observe reduced variation in highland bees.

**Fig 2:**
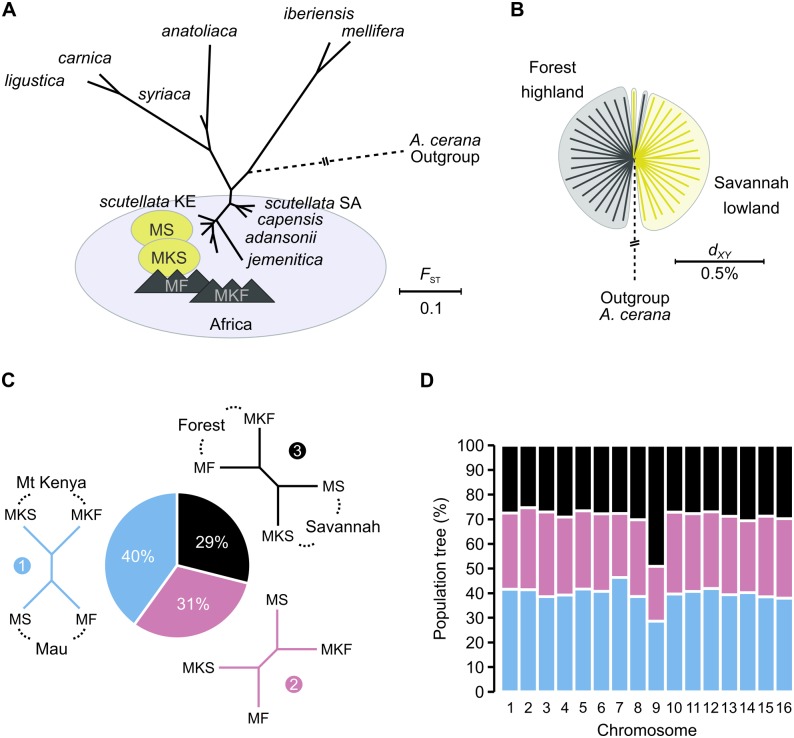
Population interrelationships. (A) Neighbor joining-tree inferred from pairwise *F*_ST_-distances between the four Kenyan population samples and previously sequenced honey bee populations from Africa (inside ellipse), the Middle East (*syriaca*; *anatoliaca*) and Europe (*ligustica*; *carnica*; *iberiensis*; *mellifera*). KE = Kenya. SA = South Africa. The position of the Eastern honey bee *A*. *cerana* used as outgroup is indicated with dashed line (not drawn to scale). (B) Interrelationships and divergence between highland and lowland bees as inferred from whole-genome estimation of the average pairwise genetic distances (*d*_*XY*_) between all specimens. (C) The proportion of alternative population interrelationships inferred from 10 kbp non-overlapping segments across the genome. Topology nr. 1 groups populations by location whereas nr. 3 groups them by environment. (D) Results from (C) subdivided by chromosomes. Color codes as in (C).

The hypothesis also predicts that highland populations share a common ancestral population and evolutionary history separate from other bees [31]. Accordingly, we should expect highland genomes to diverge from lowland genomes. We therefore calculated genome-wide *F*_ST_ between populations using the Reynolds *et al*. estimator [41]. *F*_ST_ between the Mt Kenya and Mau *monticola* and *scutellata* populations range between 0.05 and 0.068 and they all group with other Kenyan and African bees (neighbor-joining tree; Fig 2A; Table 1). Among the Kenyan bees, the *monticola* populations cluster on a short separate branch in the population tree (Fig 2A). Likewise, average pairwise genetic distances (*d*_*XY*_) split *monticola* and *scutellata* samples into different groups (Fig 2B). While this could indicate limited degrees of independent evolutionary history, we note that the excess distance between *monticola* and *scutellata* samples is very small: *d*_*XY*_ is only 1.02x higher between any random pair of highland and lowland samples compared with samples drawn within either habitat. We thus find that *monticola* populations do not diverge strongly from other Kenyan bees and that highland and lowland bees appear to be nearly undifferentiated.

It should be noted that all Kenyan populations cluster to the exclusion of the Nigerian *adansonii* and South African *scutellata* and *capensis* populations (the “sub Saharan” bees from [25]). Some of this increased divergence may be artificial and result from technical differences in short-read sequencing and mapping technologies used to assemble the extended dataset (Illumina paired-end reads+BWA herein and in [37] vs SOLiD fragments+Lifescope in [25]). For instance, the mean *F*_ST_ between the four Kenyan populations (Illumina) and the three European M-group (SOLiD) populations is 0.392, whereas it is 0.358 between the three African populations previously sequenced on SOLiD and the same European populations. We therefore estimate the magnitude of this “technology-bias” to be an increase in divergence of 9%, assuming that the distances should be the same. However, this bias does not affect our comparisons of the highland and lowland populations, which were all sequenced on the same technology and processed identically.

To determine the relationship between the closely related highland forest and lowland savannah bees, accommodating the possibility of contradictory genealogies across the genome, we inferred *F*_ST_ and the corresponding population interrelationships in 10 kbp segments. In contrast to the whole-genome signal, we found that the most common pattern of relatedness in the genome groups populations by locality (40% of windows; Fig 2C). The pattern that groups populations by habitat is the least common (29%). By partitioning the inference by chromosome, we further found that the latter pattern is recovered at approximately the same frequency on all chromosomes (25%–31%) except on chromosome 9, where it is significantly enriched (49%; p<10^−5^; Fisher’s exact test; Fig 2D). The major pattern of relatedness across the genome is therefore consistent with exchange of genetic material between local highland and lowland populations, whereas signals that cluster the populations by environment are restricted to a smaller proportion of the genome. Taken together, these analyses suggest that the extant populations of highland and lowland honey bees have the same evolutionary origin and are not isolated from each other. Our results therefore disagree with the mountain refugia hypothesis.

### Two distinct regions segregate between highland and lowland populations

**Fig 3:**
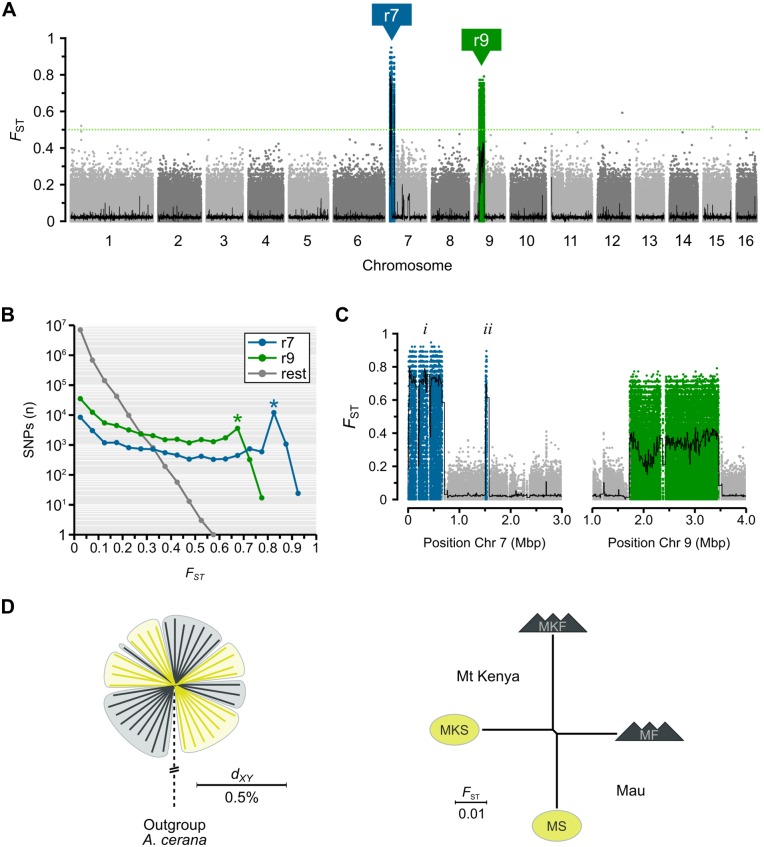
Genome-wide and localized divergence between highland and lowland populations. (A) Genome-wide plot of allele frequency differences (*F*_ST_) of every nuclear SNP (n = 8,021,515) segregating between highland bees (n = 20) and lowland bees (n = 19). Divergent regions r7 (chromosome 7; blue) and r9 (chromosome 9; green) contains nearly all *F*_ST_>0.5 SNPs (n = 24,441). Black line indicates overall *F*_ST_ across 10 kbp non-overlapping windows. (B) Number of SNPs subdivided by *F*_ST_ (0.05 bins) for r7, r9 and the rest of the genome. Stars indicate high-F_ST_ intervals with unexpectedly large numbers of SNPs. (C) Magnified view of the r7 block (left; including sub-parts *i* and *ii*) and r9 (right). Window-based *F*_ST_ as in (A). (D) Interrelationships and divergence between highland and lowland bees after exclusion of r7 and r9 SNPs. NJ-tree to the left based on average pairwise genetic distances (*d*_*XY*_) between all specimens. NJ-tree to the right inferred from *F*_ST_ between the four populations.

Genetic diversity is nearly undifferentiated between highland and lowland populations. Nevertheless, highland genomes appear to contain a small set of loci that are different from lowland genomes. These are putative targets of natural selection. Variants that are shared between the geographically separated highland populations but absent in local and closely related lowland populations could be associated with adaptation to high altitudes. We calculated *F*_ST_ (Weir-Cockerham estimator [42]) for every SNP segregating between highland samples and lowland samples in order to produce a high resolution map of differentiation across the full dataset (~29 bp/SNP) and detect such loci. The result corroborates the whole-genome estimates above. Divergence is low across the genome: genome-wide *F*_ST_ is only 0.036 and 7.7 million (97%) of SNPs have *F*_ST_<0.1 (Fig 3A and 3B). The striking exceptions are two regions on chromosomes 7 and 9, hereafter called “r7” and “r9”. Out of the 24,445 SNPs that segregate with *F*_ST_>0.5, only 4 occur on other chromosomes or outside of these regions (Fig 3A). The same divergent regions are identified when the two highland/lowland population pairs are analyzed independently (S1 Fig). A strong association between highland and lowland habitats and these two chromosomal regions were detected in a genome-wide association study (GWAS) using PLINK, where SNPs within the r7 and r9 regions are clear outliers from the expected distribution of allele frequency differences between two groups as indicated by a Q-Q plot (S2 Fig).

We delineated the r7 and r9 regions by their first and last SNPs with *F*_ST_>0.5 (Table 2), respectively. r7 bridges across four scaffolds and is composed of two blocks that together span 0.573 Mbp close to the start of chromosome 7 (*i* and *ii*; Fig 3C). Although these blocks are separated by almost 0.9 Mbp and appear to be discontinuous, we suspect that the second smaller block is located on a misoriented scaffold in an ambiguous region of the current assembly. This scaffold (7.5) is minus oriented in the reference genome, whereas upstream scaffolds have unknown orientation. The high similarity in sample genotypes between the two blocks, as well as their shared gene family components (see below), suggest that it should be reoriented. r9 spans 3 scaffolds and 1.639 Mbp on chromosome 9 (Fig 3C). *F*_ST_ between highland and lowland populations is ~0.7 and ~0.3 across the r7 and r9, respectively (Fig 3C; Table 2). They contain 111,161 SNPs in total, representing only 1.39% of the data, yet exclusion of these SNPs alone removes the split between highland and lowland samples and shifts the interrelationships of the four populations from clustering by environment to cluster by locality (Fig 3D). It is therefore clear that these narrow but divergent regions stand out against the genomic background and influence the analyses of the genome-wide interrelationships (see Fig 2B above).

**Table 2:** Differentiated regions on chromosomes 7 and 9.

Region	Chrom	Scaffolds	First SNP *F*_ST_>0.5 (bp)	Last SNP *F*_ST_>0.5 (bp)	Length (bp)	All (n)	*F*_ST_>0.5 (n)	*F*_ST_
r7	7	3 (7.1, 7.2, 7.3)	11,056	662,713	551,658	31,975	15,404	0.707
	7	1 (7.5)	1,511,853	1,533,141	21,289	1,471	597	0.639
r9	9	3 (9.5, 9.6, 9.7)	1,729,619	3,468,706	1,639,088	77,715	8,440	0.332

The differentiated regions are extended non-recombining haplotypes

The distinct blocks of highly differentiated SNPs with clear boundaries are suggestive of the presence of non-recombining haplotypes with high levels of divergence between them (Fig 3A). These patterns are unlikely to result from selective sweeps, which would be expected to result in a gradual decay of LD over shorter genetic distance. r7 contains 12,042 SNP_S_ with *F*_ST_ of 0.8–0.85 but only 2,866 SNPs with *F*_ST_ of 0.5–0.8 (Fig 3B). Likewise, r9 contains 3,587 SNPs with *F*_ST_ of 0.65–0.7 and similarly to r7, no individual bin with *F*_ST_>0.5 contains more SNPs than the 0.65–0.7 bin in the region (Fig 3B). The regions therefore appear to be enriched for SNPs at a particular high *F*_ST_ bin, indicating strong association between the segregating variants. One explanation for these patterns is that the haplotypes represent structural polymorphisms, such as inversions that prevent recombination occurring between them.

**Fig 4:**
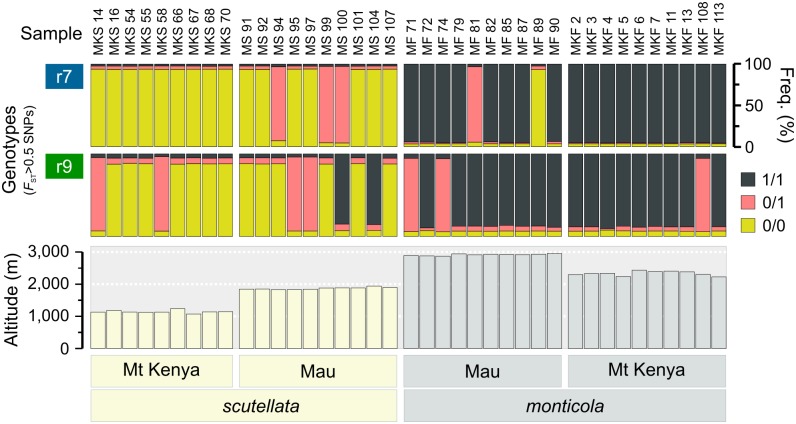
The distribution of highland and lowland haplotypes as inferred from genotypes at divergent SNPs. The proportion of genotypes where a sample is either homozygous for the honey bee reference sequence allele (0/0), homozygous for the non-reference allele (1/1) or heterozygous (0/1) is shown for every sample at both the r7 (n = 16,001) and r9 blocks (n = 8,440). Bar plots indicate the proportions of each genotype for all divergent SNPs (*F*_ST_>0.5). Bottom panels indicate altitude, location and classification of all Kenyan samples.

To further characterize r7 and r9, we counted the genotypes of every sample at all divergent SNPs (*F*_ST_>0.5; Table 2). Worker honey bees are diploid and at every such SNP, a sample can therefore be homozygous for the reference sequence allele (0/0), homozygous for the non-reference allele (1/1) or heterozygous (0/1). The reference sequence is derived from a US managed population and matches the lowland haplotype [43]. Across both regions, we found that 1/1 genotypes are significantly more frequent in highland bees than in lowland bees: 83.7% (n = 267,947) vs. 2.7% (n = 8,484) in r7 and 74.7% (n = 126,136) vs. 13.4% (n = 21,526) in r9, respectively, showing that highland bees have haplotypes that are strongly enriched for non-reference variants (p<10^−5^ for both regions; Fisher’s exact test; Fig 4). Notably, we detected several samples that appear to be nearly completely heterozygous across either region: these are heterozygous for >88% of genotypes (Fig 4). We detected a few outlier samples that are homozygous for the opposite haplotype compared to the majority of samples from either environment. The same samples are heterozygous or atypical at the two physically linked r7*i* and r7ii sub-blocks on chromosome 7 (S3A Fig), supporting the idea that they are closely located on the chromosome. This pattern pertains to other samples at the independently transmitted chromosome 9. We performed principal component analyses (PCAs) using the multidimensional scaling algorithm implemented in PLINK [44] to further evaluate these divergence patterns. The PCA was carried out for all SNPs within and outside of the r7 and r9 regions, respectively. In accordance with the *F*_ST_-based analyses, we found that divergence between highland and lowland samples was much higher at r7 and r9 than across the rest of the genome and that outlier and heterozygous samples clustered as predicted (S4 Fig). Honey bees have among the highest recorded recombination rates of any animal [45]. Continuous megabase-scale heterozygosity suggests that they have both a lowland and highland haplotype and that meiotic recombination between them is greatly suppressed.

The r7 highland haplotype (r7h) is completely fixed in the Mt Kenya highland samples and absent from the Mt Kenya lowland population, whereas we detect heterozygous samples or outliers in the Mau populations (Fig 4). The r9 highland haplotype (r9h) follows similar, albeit less extreme segregation. We estimate the average population frequencies of both r7h and r9h to be 93% across highland bees and 8% or 21% across lowland bees, respectively. Haplotype frequencies are strongly associated with environment (p<10^−5^ for both regions; *χ*^2^ test), corresponding to *F*_ST_ values of 0.832 at r7 and 0.682 at r9.

We performed coalescent simulations in order to determine the probability that the extreme differences in haplotype frequencies we observe between populations could occur in the absence of natural selection on these regions. We used ms [46] to model the evolution of a highland and lowland population and test this alternative scenario. We adopted a basic split model without subsequent gene flow between descendant populations and without recombination. Inclusion of these processes would homogenize genetic variation between the descendant populations. We applied population demographic parameters inferred from the data to model the split (see Methods) to simulate the evolution of 1 million independent loci across the genome using the same sample size as in our dataset (20 vs. 19 diploids). We then estimated *F*_ST_ between populations using the same methods with the empirical dataset. The split was inferred to have occurred 28,410 generations ago and the average divergence between simulated populations was very close the empirical data (0.037 vs 0.036). However, *F*_ST_ values as high as those observed for the r7 and r9 haplotypes (r7: *F*_ST_ = 0.832; r9: *F*_ST_ = 0.682) were never observed in the simulated data, where the most divergent locus had *F*_ST_ = 0.655 (S5 Fig). This indicates that such levels of divergence between two populations are highly unlikely to occur by drift alone under this scenario.

It is also important to note that we observe similarly extreme levels of divergence at the r7 and r9 loci in two independent highland/lowland comparisons (Mau forest vs. savannah and Mt. Kenya forest vs. savannah). There can be no direct contact between the two highland populations due to their geographic isolation, but gene flow can occur between them via the lowland populations, where frequencies of the highland haplotypes are very low. The pattern we observe, where the same haplotype variants at two loci are associated with the highland habitat in two independent comparisons is therefore indicative of selection favoring these haplotypes in highland environments. The possibility that these patterns could occur in the absence of selection can be ruled out.

### Segregation patterns on unmapped fragments

About 13% (29 Mbp) of the honey bee reference genome is not placed on any chromosome. We assessed these sequences separately and detected 982 additional SNPs with *F*_ST_>0.5, distributed across 31 scaffolds/contigs (S2A Table). We scanned these SNPs for genotype and haplotype patterns consistent with those in r7 and r9 (Fig 4). We find that 30 of them can be assigned to either r7 or r9 based on the pattern of segregation at the SNPs: 16 fragments spanning 28.6 kbp and 682 SNPs match r7 and 14 fragments covering 62.5 kbp and 299 SNPs match r9 (S2A Table; S3B–S3D Fig). The unassigned fragment GroupUn869 contains only a single outlier SNP (*F*_ST_ = 0.52). To verify the assignments, we scanned the paired-end data for evidence of split read-pairs that could anchor the unmapped fragments to the regions using Delly2 [47] in translocation mode. All 30 fragments assigned to either r7 or r9, but not GroupUn869, contain evidence that place them within haplotype scaffolds or close to their borders (S2B Table). Taken together, the results suggest that they may belong to the two regions, possibly extending them by up to 4–5%.

### Highland haplotypes in other parts of Kenya and across the species’ range

We detect homozygosity for the highland haplotypes in the single *monticola* bee collected at Mt Elgon and sequenced by Fuller and co-workers[37] (S6A Fig), and currently the only representative from a third Kenyan mountain location. The highland haplotypes appear to be absent from coastal or desert populations of Kenya. Querying the global dataset, we do not detect the r7h haplotype in any population outside of Kenya (S6B Fig). We do however detect genotypes matching r9h heterozygosity in two savannah *scutellata* samples from South Africa (S6B Fig). These samples are heterozygous for >77% of the outlier genotypes at scaffold 9.7 and have intermediate genetic distances to the Kenyan highland haplotypes compared with South African *scutellata* homozygous lowland haplotypes (S6C and S6D Fig). Interestingly, this pattern is less clear on the upstream 9.5 scaffold, where the two samples and additional South African honey bees appear to be heterozygous for only ~50% of the outlier genotypes (S6D Fig). These results suggest the presence of r9h-like haplotypes at low frequency outside of Kenya, which may include additional structural diversity.

### Highland and lowland haplotypes are highly diverged

**Fig 5:**
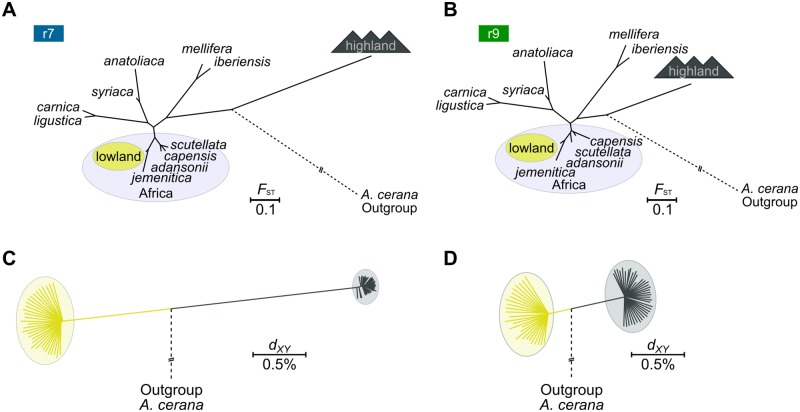
Haplotype interrelationships and divergence. (A) NJ-tree showing the interrelationships between Kenyan highland and lowland r7 haplotypes and the haplotype diversity of other honey bee populations. The position of the outgroup *A*. *cerana* is indicated with dashed line (not drawn to scale). (B) The corresponding analysis of the r9 region. (C) NJ-tree inferred from pairwise genetic distances (*d*_*XY*_) between highland and lowland r7 haplotypes. Outgroup branch not drawn to scale (*d*_*XY*_ = 6.9%). (D) The corresponding analysis of the r9 region.

We assessed genetic differentiation between populations at the r7 and r9 regions. For this analysis, we first subsampled the Kenyan data to contain only the individuals that were homozygous for the major haplotype associated with either environment (Fig 4). We included sequence variation from other honey bee populations for either region in order to analyze the haplotypes in the context of global haplotype diversity within the species. For both regions, we find that the Kenyan lowland bees have haplotypes that are typical for African honey bees (*F*_ST_<0.10 against other African bees), while the highland haplotypes diverge strongly from African and other subspecies (*F*_ST_>0.5; Fig 5A and 5B; Table 3). Other population interrelationships are consistent with the whole-genome analyses above and previous results (Fig 2A above; [25])

**Table 3:** Divergence and diversity.

Haplotype		Differentiation from other populations (*F*_ST_)				Haplotype diversity			Haplotype divergence (intergenic variants)	
Label	Environment	A^1^	O^2^	C^3^	M^4^	*π (%/bp)*	*θ*_w_ (%/bp)	*N*_E_	*d*_*XY*_ h vs l (%/bp)	Split (mya)
r7h	Highland	0.88	0.91	0.93	0.92	0.20	0.17	101,962	3.34	3.17
r7l	Lowland	0.10	0.26	0.36	0.34	0.69	0.77	500,253		
r9h	Highland	0.54	0.64	0.72	0.71	0.52	0.50	313,422	1.34	1.28
r9l	Lowland	0.07	0.24	0.39	0.37	0.72	0.80	501,515		

^1^A = Africa (*adansonii*, *scutellata*);

^2^O = Middle East (*anatoliaca*, *syriaca*);

^3^C = Central-South Eastern Europe (*carnica*, *ligustica*);

^4^M = NW Europe (*iberiensis*, *mellifera*)

We next compared the genetic distance between the two haplotypes in order to estimate timing of their divergence. Across the full genome, *d*_*XY*_ is 0.67% between any random pair of two haploid genomes (Fig 3D; Table 3). At r7 on the other hand, divergence at non-coding sites is 3.34% (95% CIs: 2.97%–3.49%, 2,000 bootstrap replicates) between highland and lowland haplotypes, dating the split between them to about 3.2(2.8–3.3) million years ago (Fig 5C; Table 3) assuming a mutation rate of *μ* = 5.27×10^−9^ mutations per base per generation and a one-year generation time. For r9, divergence is 1.34% (95% CIs: 1.28%–1.36%, 2,000 bootstrap replicates; Fig 5D), corresponding to the haplotypes having diverged about 1.28 (1.22–1.30) million years ago. These molecular clock estimates suggest that the r7 and r9 highland haplotypes have originated independently but are both very old, possibly predating the diversification of modern honey bee populations and the colonization of their current ranges by hundreds of thousands of years [25].

### Functional characterization of genes within the highland haplotypes

The r7 region has been annotated for 38 gene accessions in the current gene set, the coding regions of which span 46 kbp (8.3% of the haplotype), whereas r9 includes 50 accessions, many of which are uncharacterized, spanning only 23 kbp of coding sequence (1.4% of the haplotype) (S3 Table). By comparing fixed variants between haplotypes against the corresponding sites in *A*. *cerana*, it is possible to estimate the number of derived changes that have occurred on each haplotype after the split from their common ancestor. We inferred that 66% of the 9,941 fixed mutations have taken place on the highland haplotype, indicating higher rates of fixation in this haplotype.

To assess functional evolution on each haplotype, we quantified the ratio between fixed non-synonymous and synonymous changes that has occurred on either haplotype since their common ancestor (S3 Table). In the r7 region, there are 560 fixed coding differences between highland and lowland haplotypes, 57% (n = 323) of which we infer to have occurred on the highland haplotype. Of the derived variants fixed on the highland haplotype, 44% of (142/323) are non-synonymous. On the lowland haplotype, only 28% (66/237) of the fixed derived variants are non-synonymous. The proportion of non-synonymous variants that are fixed on the highland haplotype is therefore significantly higher than the proportion fixed on the lowland haplotype (Fisher's exact test; p<10^−5^). In the r9 region we only detect 28 fixed mutations. Out of these, 21 have occurred on the highland haplotype. Of the derived variants fixed on the highland haplotypes, 62% (n = 13) are non-synonymous. In the r9 lowland haplotype, only 28% (2 out of 7) variants are non-synonymous. These proportions show the same trend as the r7 haplotype, although they are not significantly different. It therefore appears that highland haplotypes have accumulated non-synonymous changes at a substantially higher rate.

**Fig 6:**
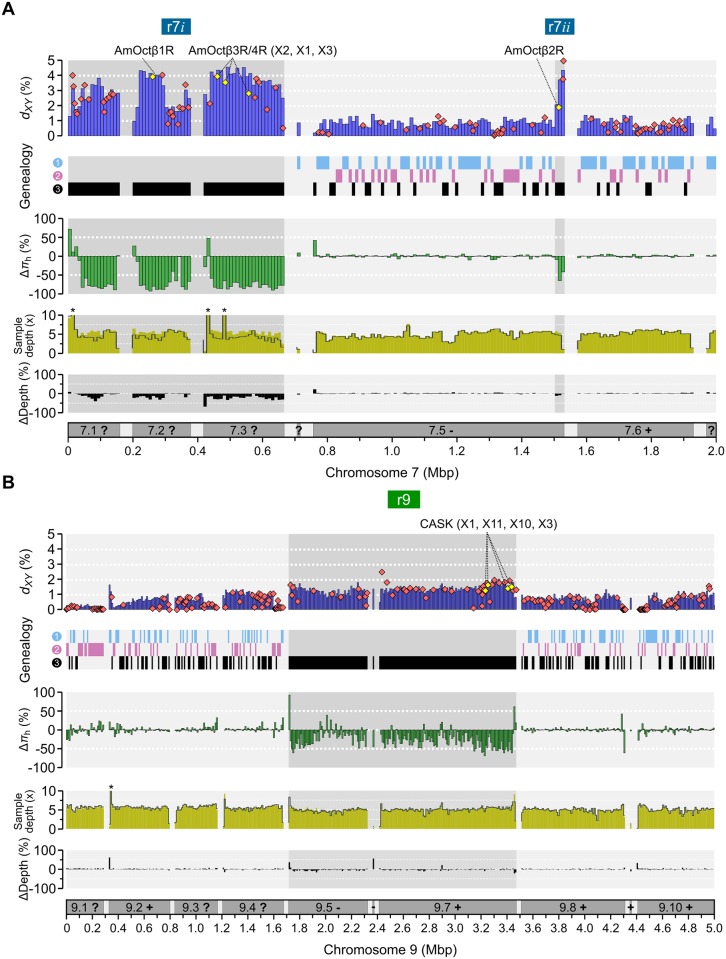
Window-based estimates of divergence and relative genetic diversity between highland and lowland haplotypes. (A) Divergence and diversity for the r7 region (r7*i*+r7*ii*; shaded) on chromosome 7 (10 kbp non-overlapping windows). From the top: i) Average pairwise genetic distances (*d*_*XY*_) between forest highland (r7h) and savannah lowland (r7l) haplotypes; diamond symbols are *d*_*XY*_ for genes (centered at gene-body mid-points); yellow diamonds are octopamine receptors; ii) four-way population interrelationships as in Fig 2C and 2D; iii) genetic diversity on r7h relative to r7l; iv) average per-haplotype sample mapping depth for r7h (grey lines) and r7l (yellow bars) with three >10× mapping peaks shared between highland and lowland haplotypes indicated with stars (per-haplotype average coverage 131×, 13× and 13×, respectively); v) the difference in mapping depth after normalizing across the whole chromosome. Scale bar indicate scaffold coordinates and orientation (? = unknown orientation) in the reference genome sequence. (B) The corresponding data for the r9 region. Shared mapping peak at 13× per-haplotype coverage indicated with star.

The two divergent regions span many divergent genes and mutations that may alter protein function, making it difficult to identify the specific targets of selection in highland bees with full certainty. Both regions contain genes that influence honey bee worker behavior that we consider to be interesting candidate genes for mediating adaptation to the montane forest habitat. The r7 region includes genes encoding four octopamine receptors: AmOctβ1R (oa2), AmOctβ3R/4R (isoforms X2, X1 & X3) and AmOctβ2R (on r7*ii*), which together contain 12 derived non-synonymous mutations in the highland haplotypes (Fig 6A; S3 Table). Octopamines are biogenic amines and essential neurotransmitters, modulators and circulatory hormones in invertebrates. They interact with specific G protein coupled receptors to increase Ca^2+^ or cAMP levels and modulate physiology and behavior in response to environmental stimuli [48,49]. In honey bee workers, octopamine increases responsiveness to sucrose and sensitivity to sensory inputs and regulates olfactory learning and memory formation [50–53]. The r9 region contains genes for encoding several isoforms of calcium/calmodulin-dependent Serine protein Kinase enzyme (CASK; LOC411347; isoforms identified with BLAST; Fig 6B). CASK interacts with a second Ca^2+^/calmodulin kinase, CaMKII, in a fundamental pathway for memory formation that is shared between humans, fly and honey bees [54–56]. We therefore hypothesize that one or both of the divergent haplotypes contain changes to genes that underlie adaptive foraging behaviors at high altitudes. Future work could focus on identifying behavioral differences in honey bees bearing contrasting haplotypes at the r7 and r9 loci.

### Genomic signal of divergent haplotype blocks is unaffected by stringency of SNP calling

We observed reduced mapping coverage in many locations across the r7 highland haplotype compared to the lowland haplotype (Fig 6A). For r7h samples, the average genotype depth was 9.2× (0.90x the genome average), with 7% of genotypes missing in the original FreeBayes SNP call. Among *F*_ST_>0.5 outlier SNPs, 2% of genotypes were missing. For r7l samples, the average genotype depth was 11.9× (1.13x the genome average), with 0.5% missing genotypes. Reduced short-read coverage can be expected due to high genetic distance between r7h and the reference genome and the difference in mapping depth is smaller for the less divergent r9 region (Fig 6B). Both regions contain a few regions with very high mapping depth that are shared between both haplotypes (Fig 6A and 6B).

Structural variants such as duplications/deletions that segregate between populations and the reference genome can alter mapping depths and result in incorrect genotyping. To assess the influence of mapping depths for the results, we analyzed a dataset of SNPs where a set of strict filters had been applied. We masked all SNPs and base pairs in the genome which had <30% or >30% read depth, compared to the average mapping depth, or where <50% of samples had been genotyped by FreeBayes. Filters were applied per environment (n = 20 highland vs. n = 19 lowland bees), for each subsample of individuals that were homozygous for either haplotype (Fig 4) or across the Kenyan bee dataset as a whole.

The filters retained ~80% of bases across the whole genome and full dataset (156 Mbp/200 Mbp) and for the r9 region (S7A Fig). At r7, 62% of the region passed the filter in the highland subsample (vs. 80% in the lowland subsample; S7A Fig). Of the original outlier SNPs with *F*_ST_>0.5, 7,205 (45%) and 5,134 (61%) were retained for r7 and r9, respectively. For both r7h and r7l subsamples, the resulting average genotype depth was ~0.95x the genome average and <0.1% of genotypes were missing. We then re-estimated and compared levels of diversity across the whole genome and the r7 and r9 haplotypes, with or without these extra filters. For the same regions, we also compared *F*_ST_ between all highland and lowland bees and re-inferred haplotypes for r7 and r9 (as in Fig 4). The results are highly congruent between datasets (S7B–S7D Fig). We therefore conclude that poorly mapped regions do not drive the patterns of diversity and divergence that we have inferred across r7, r9 or the genome.

### Haplotype breakpoint assessment

Detection of haplotype breakpoints can help to disentangle the nature of putative structural variants. In fly and mosquito, inversion breakpoints have previously been linked to crossover between repeated sequence and mobile elements [57,58]. We scanned the genomic regions around the outermost outlier SNPs of each region (Table 2) for patterns of divergent read mapping and repeated motifs. These SNPs occur close to scaffold borders and it is possible that the genome assembly is incomplete for these regions.

For r7, we find that the first outlier SNP (pos. 11,056 bp) occurs in a 500 bp region (pos. 11,000–11,500 bp) where *scutellata* reads map normally but very few *monticola* reads map (S8A Fig). The *monticola* reads that contain the first outlier SNP are truncated by BWA to ~36 bp and have mate pairs that are either unmapped or mapped to scattered regions in the genome, indicating potentially aberrant alignment. Regular *monticola* read mapping resumes at pos. ~11,420 bp. The region overlaps with repetitive sequence containing three iterations of a 176 bp AluI-like monomer with starting positions 8,814, 10,709 and 10,885, respectively (S8A Fig). This AluI-like element has been experimentally estimated to be a common repeat at honey bee telomeres [59,60]. The two latter motifs correlate with the extremely high mapping depth observed in our data (Fig 6A). The average per-sample coverage across three 100 bp windows between positions 10,700–11,000 is 8,300×, approximately 830 times the per-sample genome average (10×). This suggests that many AluI-like short reads from across the genome have been mapped to these motifs. Within the same region, we identify a 26 bp motif with high probability (“aattgataaaggaagggaggaagagg”; p<6.20 x 10^−29^) using MEME suite [61]. It is repeated 65 times between positions 8,942–10,667 and has high similarities towards a Winged Helix-turn-Helix (HTH) DNA binding protein motif likely containing the *optix* transcription factor binding site (“tgata”, relative score = 1), as predicted by JASPAR [62]. Beside their roles in transcription, proteins with HTH-binding domains are involved in recombination and may cause rearrangements[63,64].

The corresponding downstream region of r7 that may contain a haplotype breakpoint occurs around position 1,511,853, where we detect the first outlier SNP in r7*ii* (Table 2). This SNP and the subsequent divergent SNPs are located inside the third intron of the octopamine receptor AmOctβ2R gene (Fig 6; S8B Fig). Due to the strong linkage between r7*i* and r7*ii* (S3 Fig), we hypothesize that the first outlier SNP represents one end of the r7 haplotype region and that the 7.5 scaffold should be reoriented to join the octopamine receptor gene family into a continuous block. This region does not appear to contain AluI-like fragments or HTH motifs. As the7*ii* border is not as repetitive as the r7*i* upstream border, we designed a long-range PCR experiment to amplify the third intron of AmOctβ2R. If the intron contains the second breakpoint, we expect to be able to amplify the sequence in most reference-like *scutellata* samples but not in the rearranged *monticola* samples. We successfully amplified the expected 2 kbp fragments for all *scutellata* samples from Mt Kenya and Mau predicted to have the reference-like haplotype (n = 19; S8C Fig). Amplification failed for all *monticola* samples from Mt Kenya (n = 10) and four of the Mau samples predicted to be homozygous for the highland haplotypes (S8C Fig). However, amplification also worked for four Mau *monticola* samples predicted to be homozygous for the highland haplotypes. These have excess numbers of heterozygous variants for r7*ii* (S3A Fig), and are heterozygous across the test region before they switch from lowland to highland haplotypes (S8 Fig), indicating breakpoint polymorphism in this region. These results strongly suggest the presence of breakpoints within the third AmOctβ2R intron. It is possible that disruption of this octopamine receptor gene has been important for local adaptation in highland bees.

The SNP-delineated borders of the r9 region occur very close to scaffold ends (<7 kbp). In both cases, the outmost SNPs are located within 500 bp from short read alignment gaps (approx. pos. 1,729,290 and 3,468,837) that are shared between highland and lowland bees, suggesting that the data may be incomplete for these regions and that the actual r9 breakpoints are not mapped.

### No clear association with haplotype identity and body color

There is a tendency for *monticola* bees to be darker in color, whereas *scutellata* bees are more yellow, although color on its own does not distinguish highland and lowland bees and even varies within colonies [28,30]. We compared the color of our specimens with the identity of their haplotypes at r7 and r9 in order to determine if either of these loci controlled these differences in color (S9 Fig). There was no clear association with color at either of the loci. Five of the 19 specimens collected in the lowlands have uniformly dark abdomens. Of these, three are homozygous for the lowland haplotype at both loci, which would not be predicted if the highland haplotype is associated with dark abdomens. Three of the 20 highland specimens have uniformly dark abdomens. One of these is heterozygous at the r9 locus, whereas the others are fixed for highland haplotypes at both loci. It is therefore clear that there is a much stronger distinction between highland and lowland bees in the genome at r7 and r9 than there is in their color. This suggests that, while these loci may have some subtle effect on body color, they are likely associated with adaptations to highland habitats that have greater fitness consequences than differences in color.

## Discussion

This comprehensive study of the genomes of mountain honey bees in Kenya reveals novel insights into their evolutionary history and population structure. Throughout most of the genome, mountain bees are not differentiated from neighboring lowland populations, indicating that they have a recent common origin or are experiencing gene flow (Fig 2). Our results therefore contradict the mountain refugia hypothesis [29,31] but are consistent with gene flow between the highland and lowland areas [24,27]. This evidence indicates that *monticola* should not be considered a distinct subspecies but rather a regional ecotype tied to mountain forests [32,65].

We identify two haplotype blocks, r7 and r9 (on chromosomes 7 and 9, respectively), which segregate strongly between highland bees at two distinct localities and neighboring lowland bees (Fig 3). These loci are extreme outliers in terms of levels of genetic differentiation between populations, and compared to the rest of the genome and to coalescent simulations (Fig 3, S2 and S5 Figs). These blocks likely represent chromosomal rearrangements such as inversions that provide the basis for adaptation to the mountain forest environment. We find divergence between the haplotypes at r7 and r9 to be extremely high, suggesting that they began diverging much earlier than the split between extant lineages of honey bees. We hypothesize that candidate genes within these regions that have effects on foraging behavior could provide the molecular basis for local adaptation to montane forest habitats.

### Inversion polymorphisms likely govern local adaptation in mountain honey bees

The presence of long genomic blocks with high divergence between highland and lowland populations with distinct boundaries indicates that the regions harbor two distinct haplotypes. This is further supported by analysis of patterns of segregation of highly differentiated SNPs in this region among individuals, which made it possible to identify individuals that were homozygous or heterozygous for the two diverged haplotypes (Fig 4). These patterns are indicative of a form of balancing selection, where haplotypes are locally adaptive coupled with repression of interallelic exchange of genetic material through recombination between haplotypes. The most likely mechanism to prevent recombination that would lead to the observed pattern of diverged haplotypes, is a structural rearrangement such as an inversion.

Only one putative inversion breakpoint (on the r7 haplotype) mapped to known sequence in the honey bee genome assembly. We are able to amplify sequence across this breakpoint in lowland bees, but not in most highland bees, consistent with an inversion. We infer that the other breakpoint for the r7 haplotype is located close to the end of chromosome 7. There are many Alu1-like monomers and a repeated helix-turn-helix motif in the vicinity of this region. The Alu family of mobile element has previously been associated with chromosomal rearrangements in mammals [66]. It is possible that Alu-like repeats cause chromosome instabilities and rearrangements also in other taxa and have been involved in the origin of the highland haplotype in mountain bees. We are unable to identify such sequence patterns for the r9 boundaries, which correlate more strongly with scaffold borders and incomplete mapping.

There are two non-mutually-exclusive ways in which an inversion could have an effect on phenotype and fitness. The first is that the inversion mutation itself has an effect on genome function. This could be because the breakpoint disrupts a transcribed region or has an effect on gene regulation. The second is that the suppression of recombination is selectively favored because it maintains associations between co-adapted alleles. Theory suggests that inversions that capture locally adapted alleles in two populations that are connected by gene flow can quickly spread in a population [67]. Recent studies suggest that adaptation by inversions or supergenes is more common than previously thought [68,69].

There are many examples of structural inversions that are involved in local adaptation. For example in sticklebacks [5] several inversions govern local adaptation to fresh water habitats, and are present in many geographically separated regions. Adaptation to environmental clines in *Drosophila* also correlates with the frequency of cosmopolitan inversions, providing a striking example of rapid evolution [70]. It has been shown that these clines have shifted as a response to global climate change and that increased cold-tolerance may have arisen several times despite the presence of gene flow. Among butterfly species, the mimetic wing patterning of *Heliconius numata* provides a compelling example of chromosomal rearrangements that lead to co-adapted gene complexes involved in adaption and speciation [71]. A similar mechanism controls Batesian mimicry in the *Papilio* genus [72,73]. A polymorphic inversion governs worker behavior and reproductive strategies in the fire ant *Solenopsis invicta* [74,75].

We observe the same haplotypes associated with highland habitats in the two localities studied here (Mau and Mt. Kenya) and in another published dataset from Mt. Elgon [37] whereas these haplotypes appear to be rare outside of montane forest environments. This shared pattern indicates that their high frequencies in highlands is the result of selection on standing variation, rather than selection on new mutations. It is similar to the pattern of genetic adaptation observed in sticklebacks, where the same set of genetic variants, including inversions, are associated with freshwater streams and lakes across the world, but are rare in the oceans that connect them [5].

### Putative candidate genes for adaptation to montane forest

A number of genes located within the r7 and r9 regions are potential candidates for environmental adaptation in highland bees. r7 contains four out of five of the honey bee octopamine receptor genes in the genome. These are all four cAMP-inducing octopamine β-receptors (AmOctβ1R to AmOctβ4R; Fig 6A; S3 Table), whereas the single Ca^2+^ regulating AmOctαR1/oa1 is located outside of the region, on chromosome 15 [76]. Experiments with microinjections have shown that octopamine can modify neuronal responses in different neuropils of the bee brain [52]. Octopamine signaling affects complex behaviors in bees and has a key role in social division of labor and foraging [77]. Expression of octopamine receptors correlates with worker tasks and age and experimental application of the amine induces foraging in nurse bees [78–80]. Increased octopamine levels in honey bees positively affects scouting for new food sources or new nest sites [81]. In particular, octopamine plays a major role in olfactory learning and memory formation in the honey bee [50], important tasks for adapting to different environmental conditions. Interestingly, octopamine has also been shown to be important for stabilizing signaling integrity during hypoxic and thermal stress in other insects [82,83]. The four octopamine receptor genes located within r7 have many fixed differences between highland and lowland bees. Moreover, we infer that a rearrangement breakpoint is present in one of these genes that could potentially disrupt gene function (S8 Fig). We therefore hypothesize that genetic variants in the octopamine receptors, that exert their effects via mediating foraging behavior, are responsible for selective advantage of the highland version of the r7 locus in montane forests.

Another putative candidate for controlling honey bee behavior is the Ent2 gene (LOC55249) on r7, which has been associated with synaptic transmission and associative learning in *Drosophila* [84] and the Ca^2+^/calmodulin-dependent Serine protein Kinase (CASK) isoforms encoded by genes at one edge of the r9 region (Fig 6B; S3 Table). CASK acts together with CaMKII to affect long-term memory formation in honey bees [56,85]. Differences in these genes could contribute to foraging performance in highland bees. However, we cannot rule out the possibility that the divergent haplotypes have broader functional implications as they include changes to genes with *Drosophila* orthologues involved in regulating chromatin (transcriptional activator protein Pur-β; LOC72639; r7), lipid (calcium-independent phospholipase A2-gamma-like; LOC726656; r7) and polypeptide-folding functions (prefold in subunit 5-like; LOC411936; r9) as well as muscle development (myosin-2 heavy chain; LOC100576864; r9) [86]. Any of these could be important for adaptation, either independently, or as part of a co-adapted supergene complex, evolving in concert with other genes.

In addition to adaptive sequence evolution, highland haplotypes appear to have been affected by more genetic drift than lowland haplotypes. First, the levels of genetic variation are much reduced on the highland haplotypes compared to the lowland haplotypes (Table 3; Fig 6): by 76% for r7h vs r7l and by 28% for r9h vs r9l. Reduced diversity among highland haplotypes likely equates to lower *N*_E_ in these haplotypes compared to lowland haplotypes, probably due to them being more geographically restricted. Second, highland haplotypes have accumulated considerably more derived non-synonymous variants than lowland haplotypes (S3 Table), which may indicate accumulation of slightly deleterious variants. This could reflect that *N*_E_ of the mountain haplotypes may historically have been lower due to restricted distribution to particular habitats compared to the widespread lowland haplotypes and despite prevalent gene flow across the rest of the genome.

### Ancient origin of inversions and the evolution of mountain bees

High divergence between the haplotypes at r7 and r9 suggests their origin is ancient. Divergence between haplotypes at these loci is substantially higher than divergence between the major honey bee lineages found on different continents. We estimate r9 to have evolved 1.3 million years ago (MYA) and the r7 3.2 MYA using a molecular clock. Both of these dates are considerably older than estimations of the emergence of extant populations of *A*. *mellifera* [25]. There are two main explanations for such an ancient origin. First, it is possible that the haplotypes were present in the ancestral population of *A*. *mellifera*, before the split of extant lineages. They could have been involved in local adaptation before modern lineages came to inhabit their current ranges in Europe, Africa and the Middle East. Despite phenotypic similarities to European bees [27], we have not detected the *monticola* haplotypes outside Africa (Table 3; S6 Fig). A second possibility is that the highland haplotype arose from introgression with another related species in the past. This scenario is inferred to be the case with the haplotype encompassing the *EPAS1* gene in humans, which is responsible for high altitude adaptation in Tibetans and inferred to have arisen by adaptive introgression from archaic humans to modern humans [11]. In the case of *monticola* mountain bees, a potential donor population is not known. All other *Apis* species are found in Asia and their native distributions have not overlapped with that of *A*. *mellifera* until the beginning of the 20^th^ century when *A*. *mellifera* was introduced in East Asian countries.

### Conclusion

Our studies of honey bee genomes from highland and lowland populations reveal patterns that are consistent with pervasive gene flow between them, with the exception of two large and divergent blocks on chromosomes 7 and 9. Haplotypes at these loci appear to represent long inversions that are strongly differentiated between populations from different habitats. These loci are reminiscent of supergenes that have been demonstrated to govern adaptation in several other species. Many genes within these blocks are linked to honey bee behavior. In particular, we identify a haplotype breakpoint that disrupts the transcript of an octopamine receptor, part of a family of genes involved in foraging and learning. We therefore hypothesize that these loci govern the behavioral traits that are characteristic for mountain bees and likely constitute local adaptations to the highland environment. High levels of divergence between haplotypes at both loci indicate an ancient origin, suggesting that they were involved in environmental adaptation before the dispersal of honey bees to their present geographic range.

## Materials and methods

### Sample collection and DNA extraction

Female worker honey bees from each highland locality in Kenya (eastern slope of Mount Kenya and Eastern Mau Forest) and lowland samples from neighboring locations were collected as part of a previous study [28]. The highland bees are referred to as *A*. *m*. *monticola* (hereafter *monticola*), whereas lowland bees are referred to as *A*. *m*. *scutellata* (hereafter *scutellata*). The *monticola* sampling sites were closed canopy forest areas above 2000m, whereas the corresponding lowland *scutellata* samples were collected in savannah vegetation or agricultural land surrounded by savannah vegetation (Fig 1; S1A Table). We used the Maxwell Tissue DNA Purification Kit (Promega) to extract total genomic DNA from the thorax of single honey bees, each from a different colony. Images of individual abdomens were taken using a ZEISS Stereo Microscope unit Stemi 305 with an Axiocam 105 color (Fa. Zeiss, Germany).

### Sequencing and read mapping

The 39 DNA samples were barcoded and 2x125 bp paired-end reads were sequenced on an Illumina HiSeq 2500 sequencer. Reads were mapped against v4.5 of the honey bee reference genome (Amel_4.5) [43] using the default settings in the BWA v0.7.12 aligner with the “mem” algorithm [87]. Read groups and duplicates were tagged and marked with Picard v1.118. Indel-realignment and quality score recalibration was performed with GATK v3.3.0 [88], using SNPs from Wallberg *et al*. [25]. These programs were used with default settings and parameters.

Two datasets were produced. The first dataset contained genome data only from the 39 new libraries and was used for most analyses comparing Kenyan highland and lowland bees. For the second dataset, we used 11 short read archives from [37] (Kenyan samples; NCBI project ID: PRJNA237819; Illumina reads) and 98 archives from [25] (worldwide samples; NCBI project ID: PRJNA236426; SOLiD reads) in order to expand the population sample and facilitate additional comparative analyses. The former dataset was mapped with BWA as above, whereas the latter dataset was mapped with Lifescope^™^ as in [25]. See S1B and S1C Table for detailed sample information.

### SNP calling and imputation

We called single-nucleotide polymorphisms (SNPs) across all samples using the haplotype-based variant detector Freebayes v0.9.20–16 [89] for both datasets. We used the flags “-E 0”, “-X” and “-u” to suppress construction of short multi-nucleotide haplotypes from closely positioned polymorphisms and to avoid making composite polymorphisms. We used the flag “—theta 0.008” to better match the expected population mutation rate estimated in [25], as compared to the human default value (0.001). Putative SNPs were filtered for quality by accepting only biallelic SNPs with QUAL scores >50. In addition, we removed known problematic positions where a drone closely related to the DH4 individual used to produce the reference genome had been inferred to be heterozygous in [25]. As drones are haploid, they should never be heterozygous. Such SNPs are errors that indicate problematic regions in the assembly where genotyping is unreliable. This filter removed 61,157 SNPs. The procedure is detailed in [25]. The restricted dataset (n = 39 samples) contained 8,593,016 SNPs and the expanded dataset (n = 148) included 13,672,645 SNPs. BEAGLE v3.3.2 [90] was used to impute missing variants and phase haplotypes. We used the flags “iterations = 30”, “nsamples = 10” and “lowmem = true” to increase accuracy and reduce memory usage as per the recommendations in the program manual.

### Variant annotation

Gene models provided by the latest official gene set (OGSv3.2) [43] in GFF format were used to associate SNPs with genes and annotate synonymous and non-synonymous variants in coding sequences. In order to determine gene names, orthologues and putative functions, the models were cross-referenced with genes in Amel_4.5,the NCBI Annotation Release 103 (AR103) and a comprehensive set of *Drosophila* Flybase orthologues detected in [25,86]. Custom Perl scripts were used to parse the coordinates and label SNPs. We aligned the genome sequence of the Eastern honey bee *A*. *cerana* (v1.0; from Park *et al*. [38]) against the *A*. *mellifera* genome using the whole genome synteny aligner Satsuma v1 [91]. This allowed us to: i) root phylogenetic trees; and ii) use parsimony to distinguish between ancestral and derived variants at SNPs across the divergent haplotypes. In this method, the shared allele between *A*. *mellifera* and *A*. *cerana* is taken as ancestral and the non-shared allele is taken as derived.

### Divergence estimates

We used the *F*_ST_ statistic to estimate divergence between populations from pairwise differences in allele frequencies. For individual SNPs, we used the *F*_ST_ estimator of Weir and Cockerham [42]. Outlier SNPs with high *F*_ST_ compared to the genomic background served as a basis for detecting genomic regions segregating between highland and lowland bees. We applied the Reynolds *F*_ST_ estimator [41] to produce whole-genome distance matrices between all populations, allowing us to determine whether divergent regions were associated with mutations in highland or lowland bees. Reynolds *F*_ST_ was also used to compute divergence in 10 kbp windows along the genome in order to detect local support for conflicting genealogies between Kenyan populations. These statistics were estimated using custom Perl scripts. Population interrelationships were inferred from genetic distances using the neighbour-joining algorithm [92] as implemented in PHYLIP v3.696 [93] or SplitsTree v4.14.2 [94] using default settings.

Average pairwise differences were computed to estimate per-base genetic distance between all samples or between the highland and lowland haplotypes detected on chromosomes 7 and 9. These per-base genetic distances were formulated by Nei and Li [95] and are expressed as *π* when considering nucleotide diversity within populations and *d*_*XY*_ when measured between populations or haplotypes. Divergence was calculated using custom Perl scripts. We applied a constant molecular clock to date the origin of the haplotypes from *d*_*XY*_ estimated from presumably functionally neutral variants (those located outside of gene bodies). For these calculations, we used a mutation rate *μ* = 5.27×10^−9^ mutations per base per generation, which was previously estimated from neutral divergence between *A*. *mellifera* and its sister species *A*. *cerana* [25]. We assumed one generation per year as in ref [25]. We estimated 95% confidence intervals by partitioning the haplotypes into 10 kbp windows and bootstrapping the data using per-window *d*_*XY*_ estimates (2,000 replicates per region).

We performed a genome-wide association study (GWAS) to study associations between SNPs and the environment, using lowland and highland location for each sample as the case and control phenotype (S1 Table). We used the association (—assoc) function in PLINK v1.9 [44] to implement the test and plotted the observed associations over the expected assuming a random normal distribution in a Q-Q plot using the—adjust and—qq-plot flags. We also used PLINK to make a principal component analysis inside and outside the putative haplotype inversion regions using the multidimensional scaling algorithm (—mds). The regions were defined by the intervals provided in Table 2.

### Diversity estimates

To measure genetic diversity within populations, we estimated *π* (see divergence section above) and *θ*_w_ (Watterson’s theta; the population mutation rate) per base for each population [96]. Locally reduced genetic diversity can be a signal of selection or indicate reduced effective population size at that locus. Relative levels of genetic diversity between highland haplotypes (h) and lowland (l) haplotypes on chromosomes 7 and 9 was computed using the equation:

$$\Delta \pi =\frac{\pi_{h}-\pi_{l}}{\pi_{l}}$$

Strongly negative values indicate reduced variation in highland haplotypes. We used the population mutation rate (*θ*_w_) and the honey bee mutation rate (*μ*) above to estimate effective population size (*N*_E_) using the following equation:

$$N_{E}=\frac{\theta_{W}}{3\mu }$$

Because honey bees are haplodiploid, we used the inheritance scalar of 3 rather than 4, used for diploids.

### Demographic simulations

We used the program ms [46] to simulate the neutral coalescent under a basic population split scenario under a Wright-Fisher model, assuming no gene flow, no recombination and constant population size. In this scenario, a hypothetical ancestral honey bee population split into a highland and a lowland population without secondary contact. We used realistic empirical estimates of the model parameters. We first estimated the generation time since the split. Under a model of neutral divergence of two populations from a common ancestor, it is possible to convert *F*_ST_ into an estimate of time since divergence, measured in units of scaled time, which we define as *T* = *t*/3*N*_E_, where *t* is the number of generations since the split. The factor of 3 is applicable for haplodiploids. *T* can be estimated using the following formula [41]:

$$T=\frac{-\text{ln}\left(1-F_{ST}\right)}{2}$$

The average divergence between highland and lowland honey bees (*F*_ST_ = 0.036) was used to estimate *T* as 0.01833. We next determined the number of independent loci to simulate. Excluding gaps and undetermined sites, the 16 honey bee chromosomes span 199.7 Mbp in total. Given the extremely high recombination rate across the genome [45], linkage between sites is expected to decay very rapidly and the genome should therefore contain many unlinked loci. *r*^2^ was previously estimated to decay below 0.05 at distances greater than 200 bp in African honey bees [25]. We therefore assumed that the 200 Mbp genome would contain 1 million unlinked loci. Assuming the population mutation rate *θ*_w_ to be 0.0082 per base pair, as estimated from all data (Table 1), we simulated a coalescent process across a theoretical 1 kbp locus (an arbitrarily chosen size) where *θ*_w_ is scaled to 8.2. We repeated the simulation 1 million times, instructing ms to export 1 segregating site (SNP) per replicate. We sampled 40 chromosomes for one descendant population (n = 20 diploid workers) and 38 chromosomes for the other (n = 19 diploid workers). Since the coalescent tracks events back in time, the model is implemented using a population join (-ej flag) rather than a population split. The simulation was performed with a single command:

ms 78 1000000 -t 8.2 -I 2 40 38 -ej 0.01833 2 1 -s 1 > ms.simulation.data

Here, we specify to sample 78 chromosomes, repeat the analysis 1 million times, use the 1 kbp scaled mutation rate of 8.2 (-t 8.2), specify that the chromosome count for two populations is 40 and 38, respectively (-I 2 40 38), model a population join between population 2 and 1 at generation time 0.01833 (-ej 0.01833) and export one segregating locus per replicate (-s 1). This produced 1 million loci for which we calculated Weir and Cockerham *F*_ST_ individually, as for the empirical data. We produced a distribution of these simulated estimates by partitioning them into *F*_ST_ bins of 0.01.

### Haplotype breakpoint assessment

Distal outlier SNPs were used delineate haplotypes in each divergent region. Read mapping was manually inspected around these coordinates using Tablet 1.16.09.06 [97]. Repetitive element motifs in the haplotype breakpoint region on chromosome 7 were detected with the BLAST [98] and MEME [61] web services.

Some unmapped fragments (aggregated into virtual chromosome GroupUN) had outlier SNPs and genotype patterns consistent with those detected at the divergent haplotypes. In order to collect additional lines of evidence that those candidate fragments may belong to these regions, we performed split-read and paired-end analyses delly2 [47] in translocation mode (-t TRA) for the GroupUN and chromosome 7 and 9 BAM files. The program was used with default settings and the output VCF file was parsed for links between unmapped fragments and either region.

In order to test a breakpoint experimentally, oligonucleotides were developed flanking the putative breakpoint located within Octβ2R (GB49696) in chromosome 7 between positions 1,511,194 to 1,513,199. Oligonucleotide Ex3in_Ex4_1fw TTTTCTTCTCCCCCTTCTTTTC and Ex3inEx4in_1rev TTCCACTATAACCGCTTTTCC were used in a standard PCR reaction setup using high-fidelity Q5 Taq polymerase (NEB Biolabs, UK) and the following cycle conditions: 98°C 120 sec, 33x 98°C 30 sec, 58°C 25 sec, 72° 90 sec and 72°C 4 min. PCR fragments were size separated on a 1.3% agarose gel (0.5x TBE) on 140 voltage for 2.5 h in 0.5x TBE buffer. Sequence information of a subset of these PCR fragments were obtained following subsequent standard cloning procedure with pGEM-T vector system (Promega, Germany) and double strand sequencing of clones (GATC Biotech, Konstanz, Germany).

## Supporting information

S1 Fig Allele frequency differences between population pairs. (A) Genome-wide plot of allele frequency differences (*F*_ST_) of every nuclear SNP segregating between Mount Kenya highland bees (n = 10) and lowland bees (n = 9). Divergent regions r7 (chromosome 7; blue) and r9 (chromosome 9; green). Black line indicates overall *F*_ST_ across 10 kbp non-overlapping windows. (B) Corresponding contrast for Mau highland bees (n = 10) and lowland bees (n = 10).
(TIF)

S2 Fig Quantile-quantile plot of a genome-wide association study between SNPs and highland and lowland habitats. SNP *p*-values associated with the observed (y-axis) and the expected (x-axis) distribution of allele frequency differences between highland bees (n = 20) and lowland bees (n = 19). Blue line indicates the distribution where observed data equals expected data (y = x) under ideal assumptions of no population stratification.
(TIF)

S3 Fig Distribution of haplotypes as inferred from divergent SNP genotypes (n) across r7i, r7ii and the unplaced scaffolds. (A) Genotype and haplotype distributions on r7i and r7ii at SNPs that diverge between highland and lowland populations. At every genotype, a sample can be homozygous for the reference allele (0/0), homozygous for the non-reference allele (1/1) or heterozygous (0/1). Bar plots indicate the proportions of each genotype for all divergent SNPs (*F*_ST_>0.5). Sample order as in Fig 4. (B) 16 unplaced scaffolds have similar genotype and haplotype distribution to r7. Symbols as in (A). (C) 14 scaffolds have similar genotype and haplotype distribution to r9. Symbols as in (A). (D) One scaffold without similarity to either r7 or r9. Symbols as in (A).
(TIF)

S4 Fig PCA clustering of samples using PLINK multidimensional scaling. (A) Clustering based on all SNPs outside of the r7 and r9 regions. (B) Clustering in the r7 region on chromosome 7. Yellow circle indicates samples that are homozygous for the lowland haplotype. Grey circle indicates samples that are homozygous for the highland haplotype. Pink circle indicates samples that are heterozygous. Heterozygous samples and outlier samples with the opposite haplotype compared to the expected are labeled. (C) Clustering in the r9 region on chromosome 9. Symbols as in B.
(TIF)

S5 Fig *F*_ST_ distribution produced by coalescent simulations of a population split. 1 million simulated SNPs were binned according to 0.01 intervals. Blue area represents SNP *F*_ST_ distribution (y1-axis). Red line is the cumulate proportion of SNPs (y2-axis). Black dots indicate the proportion (p) of SNPs above an *F*_ST_ threshold (p = 0.05 is the top 50,000 SNPs; p = 0.01 top 10,000 SNPs; p = 0.001 top 1,000 SNPs; p = 0.0001 top 100 SNPs; p = 0.00001 top 10 SNPs; p = 0.000001 top 1 SNP). Blue and green markers indicate the respective haplotype frequency differences at r7 and r9.
(TIF)

S6 Fig Worldwide distribution of highland and lowland haplotypes. (A) Haplotypes detected for the 11 samples sequenced by Fuller et al. Color codes and bottom panels as in Fig 4. Sample order as in S1B Table. (B) A global sample of honey bees from [25]. Symbols as in Fig 4. Sample order as in S1C Table. (C) The r9 region for the African samples and subdivided for the two main scaffolds (scaffold 9.5; n = 2,231 SNPs; scaffold 9.7; n = 6,208 SNPs). (D) Divergence (*d*_*XY*_) between the two South African (SA) *scutellata* samples that appear to be heterozygous for the r9h highland haplotype (scu_3 and scu_5) and the Kenyan (KE) bees that are either homozygous for r9h (upper plot) or r9l (lower plot). *d*_*XY*_ between South African (SA) *scutellata* samples homozygous for the lowland haplotype and the same Kenyan bees indicated in yellow. *d*_*XY*_ within either group of Kenyan bees indicated in black.
(TIF)

S7 Fig Extended filtering and quality control. (A) Proportion of retained sites across the genome or divergent regions r7 and r9 after stringent filtering for mapping depth and sample coverage (see Results section for filters; r7h = r7 highland haplogroup; r7l = r7 lowland haplogroup; r9h = r9 highland haplogroup; r9l = r9 lowland haplogroup). (B) Average genetic diversity across the regions in A based on all data or after filtering. (C) *F*_ST_ between highland and lowland bees based on all data or after filtering. (D) Haplotype patterns for r7 and r9 after filtering (as compared to Fig 4).
(TIF)

S8 Fig r7 haplotype breakpoints. (A) Tablet visualization of read mapping across the putative breakpoint at the start of the r7 haplotype using. Reads from bees with r7 lowland haplotypes are yellow. Reads from bees with r7 highland haplotypes are grey. Reads from heterozygous samples are pink. Light blue box indicates spuriously mapped region in highland samples and contains the first outlier SNP at position 11,056 bp. Dashed blue box indicates a potential breakpoint region where no full read pairs from highland samples map. Red bands indicate location of AluI-like elements detected with BLAST. (B) SNPs across the octopamine receptor gene AmOctβ2R (GB49696). Gene body depicted in green (thin lines = introns; thick lines = exons). Dashed blue line indicates a putative breakpoint and amplification target in intron 3. Triangles indicate points where four Mau (MF) samples switch from being heterozygous for highland haplotypes to being homozygous. (C) Gel pictures of PCR products after attempting to amplify across the region indicated in (B).
(TIF)

S9 Fig Abdominal pigmentation in *monticola* forest highland bees and *scutellata* savannah lowland bees. Stars indicate samples with contrasting haplotypes for r7 (blue) or r9 (green) from the common haplotype in either habitat (see Fig 4). Black bars indicate individuals with back/dark pigmentation across all tergites of the abdomen.
(TIF)

S1 Table Sample locations and sequence information. (XLSX)

S2 Table Possible locations of unplaced scaffolds with outlier SNPs. (XLSX)

S3 Table Divergence and substitutions across genes in highland and lowland haplotypes. (XLSX)

## References

[1] Barrett RDH, Hoekstra HE. Molecular spandrels: tests of adaptation at the genetic level. Nat Rev Genet. 2011;12: 767–780. 10.1038/nrg3015 22005986

[2] Nachman MW, Hoekstra HE, D’Agostino SL. The genetic basis of adaptive melanism in pocket mice. Proc Natl Acad Sci. 2003;100: 5268–5273. 10.1073/pnas.0431157100 12704245 PMC154334

[3] van’t Hof AE, Edmonds N, Dalíková M, Marec F, Saccheri IJ. Industrial Melanism in British Peppered Moths Has a Singular and Recent Mutational Origin. Science. 2011;332: 958–960. 10.1126/science.1203043 21493823

[4] van’t Hof AE, Campagne P, Rigden DJ, Yung CJ, Lingley J, Quail MA, et al The industrial melanism mutation in British peppered moths is a transposable element. Nature. 2016;534: 102–105. 10.1038/nature17951 27251284

[5] Jones FC, Grabherr MG, Chan YF, Russell P, Mauceli E, Johnson J, et al The genomic basis of adaptive evolution in threespine sticklebacks. Nature. 2012;484: 55–61. 10.1038/nature10944 22481358 PMC3322419

[6] Chan YF, Marks ME, Jones FC, Villarreal G, Shapiro MD, Brady SD, et al Adaptive evolution of pelvic reduction in sticklebacks by recurrent deletion of a Pitx1 enhancer. Science. 2010;327: 302–305. 10.1126/science.1182213 20007865 PMC3109066

[7] Takuno S, Ralph P, Swarts K, Elshire RJ, Glaubitz JC, Buckler ES, et al Independent Molecular Basis of Convergent Highland Adaptation in Maize. Genetics. 2015;200: 1297–1312. 10.1534/genetics.115.178327 26078279 PMC4571994

[8] Bigham A, Bauchet M, Pinto D, Mao X, Akey JM, Mei R, et al Identifying Signatures of Natural Selection in Tibetan and Andean Populations Using Dense Genome Scan Data. PLOS Genet. 2010;6: e1001116 10.1371/journal.pgen.1001116 20838600 PMC2936536

[9] Alkorta-Aranburu G, Beall CM, Witonsky DB, Gebremedhin A, Pritchard JK, Di Rienzo A. The genetic architecture of adaptations to high altitude in Ethiopia. PLoS Genet. 2012;8: e1003110 10.1371/journal.pgen.1003110 23236293 PMC3516565

[10] Scheinfeldt LB, Soi S, Thompson S, Ranciaro A, Woldemeskel D, Beggs W, et al Genetic adaptation to high altitude in the Ethiopian highlands. Genome Biol. 2012;13: R1 10.1186/gb-2012-13-1-r1 22264333 PMC3334582

[11] Huerta-Sánchez E, Jin X, Asan, Bianba Z, Peter BM, Vinckenbosch N, et al Altitude adaptation in Tibetans caused by introgression of Denisovan-like DNA. Nature. 2014;512: 194–197. 10.1038/nature13408 25043035 PMC4134395

[12] Natarajan C, Hoffmann FG, Weber RE, Fago A, Witt CC, Storz JF. Predictable convergence in hemoglobin function has unpredictable molecular underpinnings. Science. 2016;354: 336–339. 10.1126/science.aaf9070 27846568 PMC5464326

[13] Pritchard JK, Rienzo AD. Adaptation—not by sweeps alone. Nat Rev Genet. 2010;11: 665–667. 10.1038/nrg2880 20838407 PMC4652788

[14] Huerta-Sánchez E, DeGiorgio M, Pagani L, Tarekegn A, Ekong R, Antao T, et al Genetic signatures reveal high-altitude adaptation in a set of Ethiopian populations. Mol Biol Evol. 2013; mst089. 10.1093/molbev/mst089 PMC3708501 23666210

[15] Peng Y, Yang Z, Zhang H, Cui C, Qi X, Luo X, et al Genetic Variations in Tibetan Populations and High-Altitude Adaptation at the Himalayas. Mol Biol Evol. 2011;28: 1075–1081. 10.1093/molbev/msq290 21030426

[16] Xu S, Li S, Yang Y, Tan J, Lou H, Jin W, et al A Genome-Wide Search for Signals of High-Altitude Adaptation in Tibetans. Mol Biol Evol. 2011;28: 1003–1011. 10.1093/molbev/msq277 20961960

[17] Schluter D, Marchinko KB, Barrett RDH, Rogers SM. Natural selection and the genetics of adaptation in threespine stickleback. Philos Trans R Soc B Biol Sci. 2010;365: 2479–2486. 10.1098/rstb.2010.0036 PMC2935102 20643737

[18] Savolainen O, Lascoux M, Merilä J. Ecological genomics of local adaptation. Nat Rev Genet. 2013;14: 807–820. 10.1038/nrg3522 24136507

[19] Boake CRB, Arnold SJ, Breden F, Meffert LM, Ritchie MG, Taylor BJ, et al Genetic tools for studying adaptation and the evolution of behavior. Am Nat. 2002;160 Suppl 6: S143–159. 18707473 10.1086/342902

[20] Weber JN, Peterson BK, Hoekstra HE. Discrete genetic modules are responsible for complex burrow evolution in Peromyscus mice. Nature. 2013;493: 402–405. 10.1038/nature11816 23325221

[21] Seeley TD. Honeybee Democracy. Princeton University Press; 2010.

[22] Seeley TD. The Wisdom of the Hive. Harvard University Press; 1995.

[23] Härtel S, Steffan-Dewenter I. Ecology: Honey Bee Foraging in Human-Modified Landscapes. Curr Biol. 2014;24: R524–R526. 10.1016/j.cub.2014.04.052 24892913

[24] Ruttner F. Biogeography and taxonomy of honeybees. Springer-Verlag; 1988.

[25] Wallberg A, Han F, Wellhagen G, Dahle B, Kawata M, Haddad N, et al A worldwide survey of genome sequence variation provides insight into the evolutionary history of the honeybee Apis mellifera. Nat Genet. 2014;46: 1081–1088. 10.1038/ng.3077 25151355

[26] Bussmann RW. Vegetation zonation and nomenclature of African Mountains An overview. Lyonia. 2006;11: 41–66.

[27] Smith FG. The Races of Honeybees in Africa. Bee World. 1961;42: 255–260.

[28] Gruber K, Schöning C, Otte M, Kinuthia W, Hasselmann M. Distinct subspecies or phenotypic plasticity? Genetic and morphological differentiation of mountain honey bees in East Africa. Ecol Evol. 2013;3: 3204–3218. 10.1002/ece3.711 24223262 PMC3797471

[29] Meixner M, Ruttner F, Koeniger N, Koeniger G. The mountain bees of the Kilimanjaro region and their relation to neighbouring bee populations. Apidologie. 1989;20: 165–174.

[30] Meixner MD, Sheppard WS, Dietz A, Krell R. Morphological and allozyme variability in honey bees from Kenya. Apidologie. 1994;25: 188–202.

[31] Meixner MD, Arias MC, Sheppard WS. Mitochondrial DNA polymorphisms in honey bee subspecies from Kenya. Apidologie. 2000;31: 181–190.

[32] Hepburn HR, Radloff SE, Oghiakhe S. Mountain honeybees of Africa. Apidologie. 2000;31: 17.

[33] Drescher W. Bienennutzung in Tansania. Allg Dtsch Imkerztg. 1975; http://agris.fao.org/agris-search/search.do?recordID=US201302741694

[34] Franck P, Koeniger N, Lahner G, Crewe RM, Solignac M. Evolution of extreme polyandry: an estimate of mating frequency in two African honeybee subspecies, Apis mellifera monticola and A.m. scutellata: Insectes Sociaux. 2000;47: 364–370.

[35] Hepburn HR, Radloff SE. Honeybees of Africa. Springer; 1998.

[36] Österlund E. Exploring Monticola—Efforts to Find an Acceptable Varroa-Resistant Honey Bee. Am Bee J. 1991; 49–56.

[37] Fuller ZL, Niño EL, Patch HM, Bedoya-Reina OC, Baumgarten T, Muli E, et al Genome-wide analysis of signatures of selection in populations of African honey bees (Apis mellifera) using new web-based tools. BMC Genomics. 2015;16: 518 10.1186/s12864-015-1712-0 26159619 PMC4496815

[38] Park D, Jung JW, Choi B-S, Jayakodi M, Lee J, Lim J, et al Uncovering the novel characteristics of Asian honey bee, Apis cerana, by whole genome sequencing. BMC Genomics. 2015;16: 1 10.1186/1471-2164-16-1 25553907 PMC4326529

[39] Kimura M. The Neutral Theory of Molecular Evolution: [Internet]. Cambridge: Cambridge University Press; 1983 https://www.cambridge.org/core/books/the-neutral-theory-of-molecular-evolution/0FF60E9F47915B17FFA2620C49400632

[40] Ellstrand NC, Elam DR. Population Genetic Consequences of Small Population Size: Implications for Plant Conservation. Annu Rev Ecol Syst. 1993;24: 217–242.

[41] Reynolds J, Weir BS, Cockerham CC. Estimation of the Coancestry Coefficient: Basis for a Short-Term Genetic Distance. Genetics. 1983;105: 767–779. 17246175 10.1093/genetics/105.3.767 PMC1202185

[42] Weir BS, Cockerham CC. Estimating F-Statistics for the Analysis of Population Structure. Evolution. 1984;38: 1358–1370. 28563791 10.1111/j.1558-5646.1984.tb05657.x

[43] Elsik CG, Worley KC, Bennett AK, Beye M, Camara F, Childers CP, et al Finding the missing honey bee genes: lessons learned from a genome upgrade. BMC Genomics. 2014;15: 86 10.1186/1471-2164-15-86 24479613 PMC4028053

[44] Purcell S, Neale B, Todd-Brown K, Thomas L, Ferreira MAR, Bender D, et al PLINK: a tool set for whole-genome association and population-based linkage analyses. Am J Hum Genet. 2007;81: 559–575. 10.1086/519795 17701901 PMC1950838

[45] Wallberg A, Glémin S, Webster MT. Extreme Recombination Frequencies Shape Genome Variation and Evolution in the Honeybee, Apis mellifera. PLoS Genet. 2015;11: e1005189 10.1371/journal.pgen.1005189 25902173 PMC4406589

[46] Hudson RR. Generating samples under a Wright-Fisher neutral model of genetic variation. Bioinformatics. 2002;18: 337–338. 11847089 10.1093/bioinformatics/18.2.337

[47] Rausch T, Zichner T, Schlattl A, Stütz AM, Benes V, Korbel JO. DELLY: structural variant discovery by integrated paired-end and split-read analysis. Bioinformatics. 2012;28: i333–i339. 10.1093/bioinformatics/bts378 22962449 PMC3436805

[48] Roeder T. TYRAMINE AND OCTOPAMINE: Ruling Behavior and Metabolism. Annu Rev Entomol. 2005;50: 447–477. 10.1146/annurev.ento.50.071803.130404 15355245

[49] Roeder T, Seifert M, Kähler C, Gewecke M. Tyramine and octopamine: Antagonistic modulators of behavior and metabolism. Arch Insect Biochem Physiol. 2003;54: 1–13. 10.1002/arch.10102 12942511

[50] Hammer M, Menzel R. Multiple Sites of Associative Odor Learning as Revealed by Local Brain Microinjections of Octopamine in Honeybees. Learn Mem. 1998;5: 146–156. 10454379 PMC311245

[51] Behrends A, Scheiner R. Octopamine improves learning in newly emerged bees but not in old foragers. J Exp Biol. 2012;215: 1076–1083. 10.1242/jeb.063297 22399652

[52] Erber J, Kloppenburg P. The modulatory effects of serotonin and octopamine in the visual system of the honey bee (Apis mellifera L.). J Comp Physiol A. 1995;176: 111–118.

[53] Scheiner R, Plückhahn S, Öney B, Blenau W, Erber J. Behavioural pharmacology of octopamine, tyramine and dopamine in honey bees. Behav Brain Res. 2002;136: 545–553. 12429417 10.1016/s0166-4328(02)00205-x

[54] Gillespie JM, Hodge JJL. CASK regulates CaMKII autophosphorylation in neuronal growth, calcium signaling, and learning. Front Mol Neurosci. 2013;6: 27 10.3389/fnmol.2013.00027 24062638 PMC3769642

[55] Malik BR, Gillespie JM, Hodge JJL. CASK and CaMKII function in the mushroom body α′/β′ neurons during Drosophila memory formation. Front Neural Circuits. 2013;7. 10.3389/fncir.2013.00052 PMC3608901 23543616

[56] Scholl C, Kübert N, Muenz TS, Rössler W. CaMKII knockdown affects both early and late phases of olfactory long-term memory in the honeybee. J Exp Biol. 2015;218: 3788–3796. 10.1242/jeb.124859 26486369

[57] Puerma E, Orengo DJ, Salguero D, Papaceit M, Segarra C, Aguadé M. Characterization of the Breakpoints of a Polymorphic Inversion Complex Detects Strict and Broad Breakpoint Reuse at the Molecular Level. Mol Biol Evol. 2014; msu177. 10.1093/molbev/msu177 24881049

[58] Lobo NF, Sangaré DM, Regier AA, Reidenbach KR, Bretz DA, Sharakhova MV, et al Breakpoint structure of the Anopheles gambiae 2Rb chromosomal inversion. Malar J. 2010;9: 293 10.1186/1475-2875-9-293 20974007 PMC2988034

[59] Tarès S, Cornuet JM, Abad P. Characterization of an unusually conserved AluI highly reiterated DNA sequence family from the honeybee, Apis mellifera. Genetics. 1993;134: 1195–1204. 8104160 10.1093/genetics/134.4.1195 PMC1205586

[60] Weinstock GM, Robinson GE, Gibbs RA, Weinstock GM, Weinstock GM, Robinson GE, et al Insights into social insects from the genome of the honeybee Apis mellifera. Nature. 2006;443: 931–949. 10.1038/nature05260 17073008 PMC2048586

[61] Bailey TL, Boden M, Buske FA, Frith M, Grant CE, Clementi L, et al MEME Suite: tools for motif discovery and searching. Nucleic Acids Res. 2009;37: W202–W208. 10.1093/nar/gkp335 19458158 PMC2703892

[62] Mathelier A, Zhao X, Zhang AW, Parcy F, Worsley-Hunt R, Arenillas DJ, et al JASPAR 2014: an extensively expanded and updated open-access database of transcription factor binding profiles. Nucleic Acids Res. 2013; gkt997. 10.1093/nar/gkt997 PMC3965086 24194598

[63] Harami GM, Gyimesi M, Kovács M. From keys to bulldozers: expanding roles for winged helix domains in nucleic-acid-binding proteins. Trends Biochem Sci. 2013;38: 364–371. 10.1016/j.tibs.2013.04.006 23768997

[64] Chen SH, Chan N-L, Hsieh T. New Mechanistic and Functional Insights into DNA Topoisomerases. Annu Rev Biochem. 2013;82: 139–170. 10.1146/annurev-biochem-061809-100002 23495937

[65] Kerr WE. Abejas Africanas su introduccion y expansion en el continente americano. Subespecies y ecotipos Africanos. Ind Apic. 1992;13: 12–21.

[66] Lee J, Han K, Meyer TJ, Kim H-S, Batzer MA. Chromosomal Inversions between Human and Chimpanzee Lineages Caused by Retrotransposons. PLOS ONE. 2008;3: e4047 10.1371/journal.pone.0004047 19112500 PMC2603318

[67] Kirkpatrick M, Barton N. Chromosome inversions, local adaptation and speciation. Genetics. 2006;173: 419–434. 10.1534/genetics.105.047985 16204214 PMC1461441

[68] Thompson MJ, Jiggins CD. Supergenes and their role in evolution. Heredity. 2014;113: 1–8. 10.1038/hdy.2014.20 24642887 PMC4815649

[69] Hoffmann AA, Rieseberg LH. Revisiting the Impact of Inversions in Evolution: From Population Genetic Markers to Drivers of Adaptive Shifts and Speciation? Annu Rev Ecol Evol Syst. 2008;39: 21–42. 10.1146/annurev.ecolsys.39.110707.173532 20419035 PMC2858385

[70] Bergland AO, Tobler R, González J, Schmidt P, Petrov D. Secondary contact and local adaptation contribute to genome-wide patterns of clinal variation in Drosophila melanogaster. Mol Ecol. 2016;25: 1157–1174. 10.1111/mec.13455 26547394 PMC5089930

[71] Joron M, Frezal L, Jones RT, Chamberlain NL, Lee SF, Haag CR, et al Chromosomal rearrangements maintain a polymorphic supergene controlling butterfly mimicry. Nature. 2011;477: 203–206. 10.1038/nature10341 21841803 PMC3717454

[72] Kunte K, Zhang W, Tenger-Trolander A, Palmer DH, Martin A, Reed RD, et al doublesex is a mimicry supergene. Nature. 2014;507: 229–232. 10.1038/nature13112 24598547

[73] Nishikawa H, Iijima T, Kajitani R, Yamaguchi J, Ando T, Suzuki Y, et al A genetic mechanism for female-limited Batesian mimicry in Papilio butterfly. Nat Genet. 2015;47: 405–409. 10.1038/ng.3241 25751626

[74] Keller L, Ross KG. Selfish genes: a green beard in the red fire ant. Nature. 1998;394: 573–575.

[75] Wang J, Wurm Y, Nipitwattanaphon M, Riba-Grognuz O, Huang Y-C, Shoemaker D, et al A Y-like social chromosome causes alternative colony organization in fire ants. Nature. 2013;493: 664–668. 10.1038/nature11832 23334415

[76] Balfanz S, Jordan N, Langenstück T, Breuer J, Bergmeier V, Baumann A. Molecular, pharmacological, and signaling properties of octopamine receptors from honeybee (Apis mellifera) brain. J Neurochem. 2014;129: 284–296. 10.1111/jnc.12619 24266860

[77] Johnson BR. Division of labor in honeybees: form, function, and proximate mechanisms. Behav Ecol Sociobiol. 2010;64: 305–316. 10.1007/s00265-009-0874-7 20119486 PMC2810364

[78] Reim T, Scheiner R. Division of labour in honey bees: age- and task-related changes in the expression of octopamine receptor genes. Insect Mol Biol. 2014;23: 833–841. 10.1111/imb.12130 25187440

[79] Schulz DJ, Sullivan JP, Robinson GE. Juvenile hormone and octopamine in the regulation of division of labor in honey bee colonies. Horm Behav. 2002;42: 222–231. 12367575 10.1006/hbeh.2002.1806

[80] Schulz DJ, Robinson GE. Octopamine influences division of labor in honey bee colonies. J Comp Physiol [A]. 2001;187: 53–61. 10.1007/s003590000177 11318378

[81] Liang ZS, Nguyen T, Mattila HR, Rodriguez-Zas SL, Seeley TD, Robinson GE. Molecular determinants of scouting behavior in honey bees. Science. 2012;335: 1225–1228. 10.1126/science.1213962 22403390

[82] Armstrong GAB, Meldrum Robertson R. A role for octopamine in coordinating thermoprotection of an insect nervous system. J Therm Biol. 2006;31: 149–158.

[83] Money TGA, Sproule MKJ, Cross KP, Robertson RM. Octopamine stabilizes conduction reliability of an unmyelinated axon during hypoxic stress. J Neurophysiol. 2016;116: 949–959. 10.1152/jn.00354.2016 27281750 PMC5009204

[84] Knight D, Harvey PJ, Iliadi KG, Klose MK, Iliadi N, Dolezelova E, et al Equilibrative Nucleoside Transporter 2 Regulates Associative Learning and Synaptic Function in Drosophila. J Neurosci. 2010;30: 5047–5057. 10.1523/JNEUROSCI.6241-09.2010 20371825 PMC6632785

[85] Matsumoto Y, Sandoz J-C, Devaud J-M, Lormant F, Mizunami M, Giurfa M. Cyclic nucleotide–gated channels, calmodulin, adenylyl cyclase, and calcium/calmodulin-dependent protein kinase II are required for late, but not early, long-term memory formation in the honeybee. Learn Mem. 2014;21: 272–286. 10.1101/lm.032037.113 24741108 PMC3994501

[86] Attrill H, Falls K, Goodman JL, Millburn GH, Antonazzo G, Rey AJ, et al FlyBase: establishing a Gene Group resource for Drosophila melanogaster. Nucleic Acids Res. 2016;44: D786–D792. 10.1093/nar/gkv1046 26467478 PMC4702782

[87] Li H, Durbin R. Fast and accurate long-read alignment with Burrows–Wheeler transform. Bioinformatics. 2010;26: 589–595. 10.1093/bioinformatics/btp698 20080505 PMC2828108

[88] McKenna A, Hanna M, Banks E, Sivachenko A, Cibulskis K, Kernytsky A, et al The Genome Analysis Toolkit: A MapReduce framework for analyzing next-generation DNA sequencing data. Genome Res. 2010;20: 1297–1303. 10.1101/gr.107524.110 20644199 PMC2928508

[89] Garrison E, Marth G. Haplotype-based variant detection from short-read sequencing. ArXiv12073907 Q-Bio. 2012; http://arxiv.org/abs/1207.3907

[90] Browning SR, Browning BL. Rapid and accurate haplotype phasing and missing-data inference for whole-genome association studies by use of localized haplotype clustering. Am J Hum Genet. 2007;81: 1084–1097. 10.1086/521987 17924348 PMC2265661

[91] Grabherr MG, Russell P, Meyer M, Mauceli E, Alföldi J, Palma FD, et al Genome-wide synteny through highly sensitive sequence alignment: Satsuma. Bioinformatics. 2010;26: 1145–1151. 10.1093/bioinformatics/btq102 20208069 PMC2859124

[92] Saitou N, Nei M. The neighbor-joining method: a new method for reconstructing phylogenetic trees. Mol Biol Evol. 1987;4: 406–425. 3447015 10.1093/oxfordjournals.molbev.a040454

[93] Felsenstein J. PHYLIP (Phylogeny Inference Package) version 3.6. Distributed by the author. Department of Genome Sciences, University of Washington, Seattle; 2005.

[94] Pickrell JK, Pritchard JK. Inference of Population Splits and Mixtures from Genome-Wide Allele Frequency Data. Tang H, editor. PLoS Genet. 2012;8: e1002967 10.1371/journal.pgen.1002967 23166502 PMC3499260

[95] Nei M, Li WH. Mathematical model for studying genetic variation in terms of restriction endonucleases. Proc Natl Acad Sci. 1979;76: 5269–5273. 291943 10.1073/pnas.76.10.5269 PMC413122

[96] Watterson GA. On the number of segregating sites in genetical models without recombination. Theor Popul Biol. 1975;7: 256–276. 1145509 10.1016/0040-5809(75)90020-9

[97] Milne I, Stephen G, Bayer M, Cock PJA, Pritchard L, Cardle L, et al Using Tablet for visual exploration of second-generation sequencing data. Brief Bioinform. 2013;14: 193–202. 10.1093/bib/bbs012 22445902

[98] Altschul SF, Gish W, Miller W, Myers EW, Lipman DJ. Basic local alignment search tool. J Mol Biol. 1990;215: 403–410. 10.1016/S0022-2836(05)80360-2 2231712

