# Supplementary figures and images for: Two extended haplotype blocks are associated with adaptation to high altitude habitats in East African honey bees
Source: PLoS Genet. 2017 May 25;13(5):e1006792. doi: 10.1371/journal.pgen.1006792 (PMC5444601; doi:10.1371/journal.pgen.1006792)

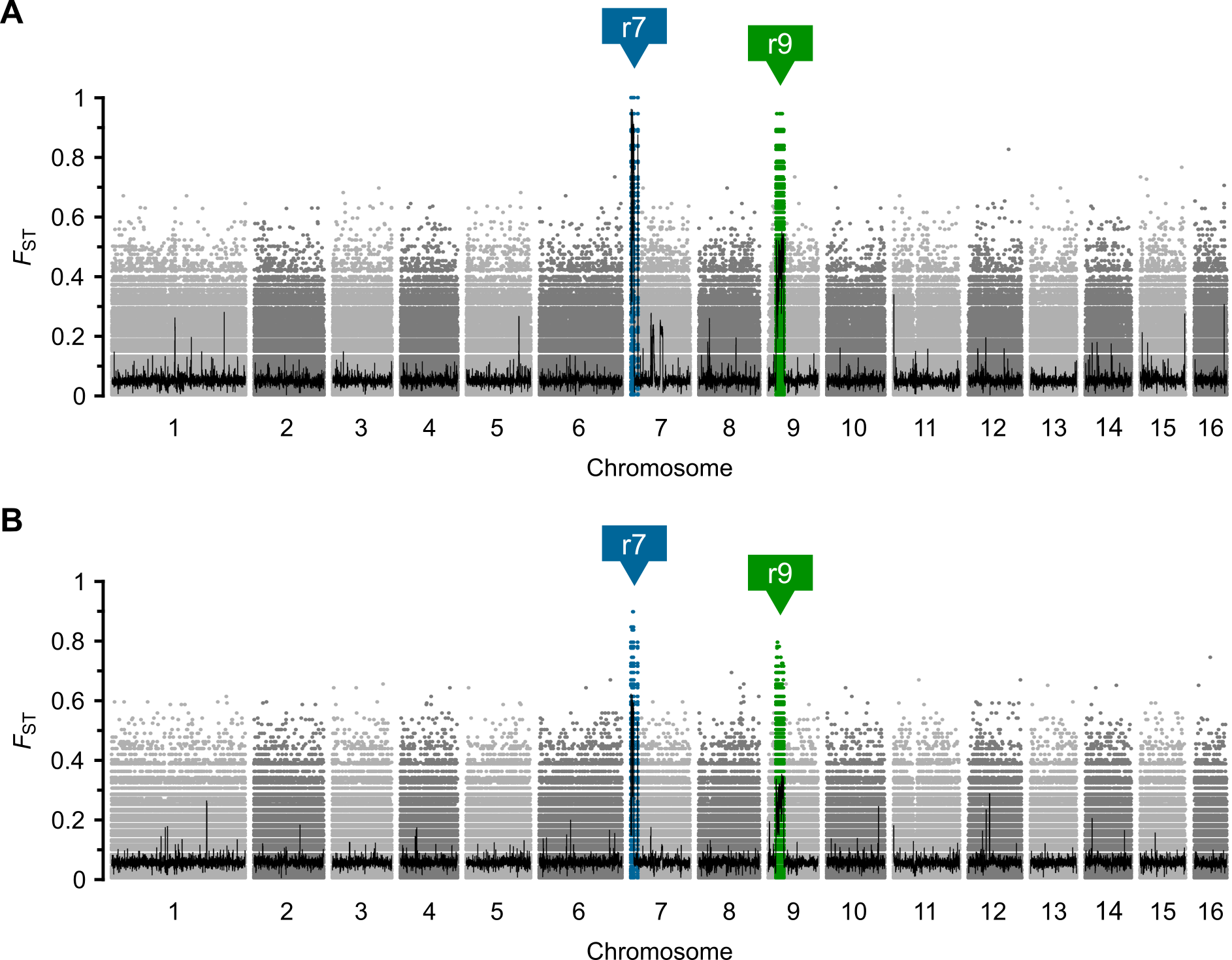

Supplement: S1 Fig — (A) Genome-wide plot of allele frequency differences (FST) of every nuclear SNP segregating between Mount Kenya highland bees (n = 10) and lowland bees (n = 9). Divergent regions r7 (chromosome 7; blue) and r9 (chromosome 9; green). Black line indicates overall FST across 10 kbp non-overlapping windows. (B) Corresponding contrast for Mau highland bees (n = 10) and lowland bees (n = 10). (TIF) [file pgen.1006792.s001.tif]

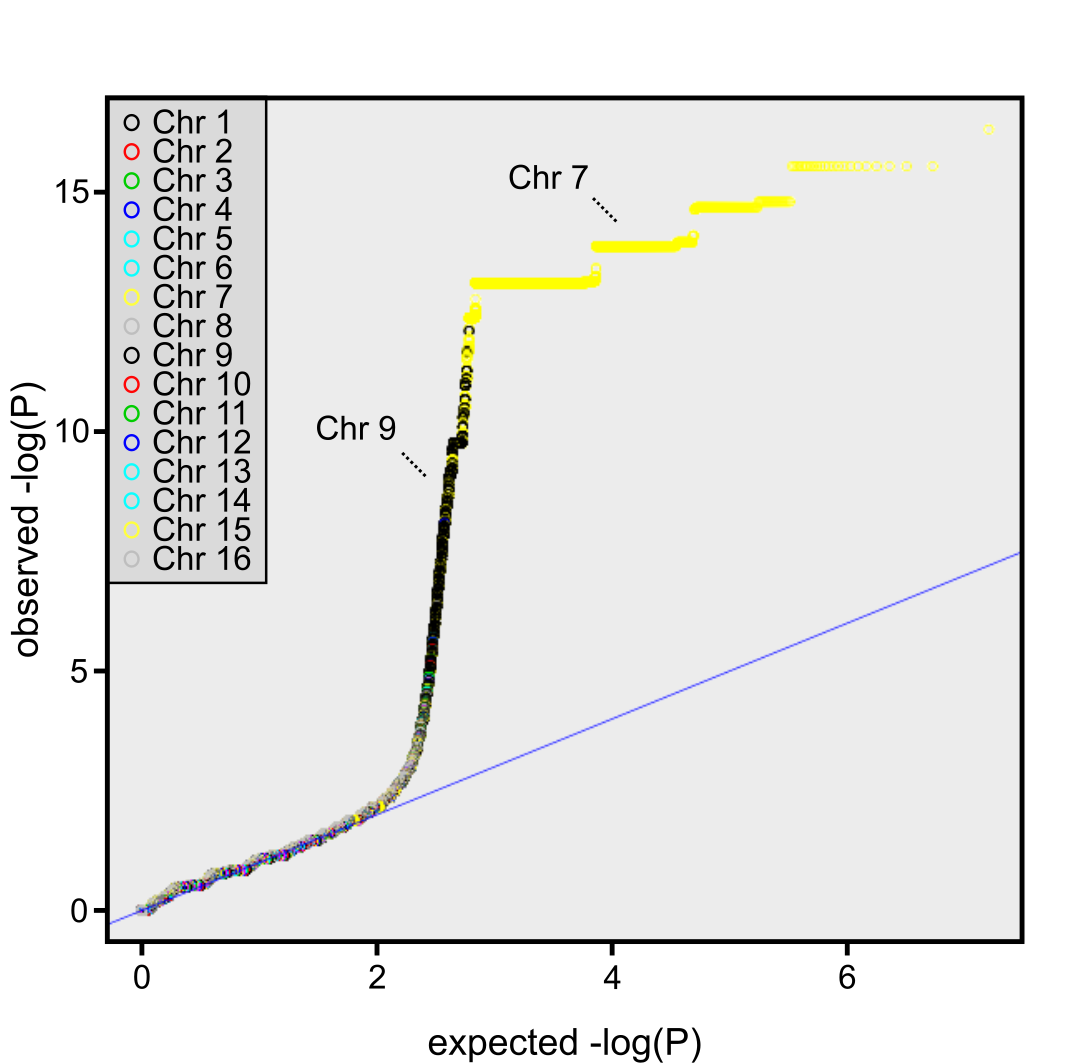

Supplement: S2 Fig — SNP p-values associated with the observed (y-axis) and the expected (x-axis) distribution of allele frequency differences between highland bees (n = 20) and lowland bees (n = 19). Blue line indicates the distribution where observed data equals expected data (y = x) under ideal assumptions of no population stratification. (TIF) [file pgen.1006792.s002.tif]

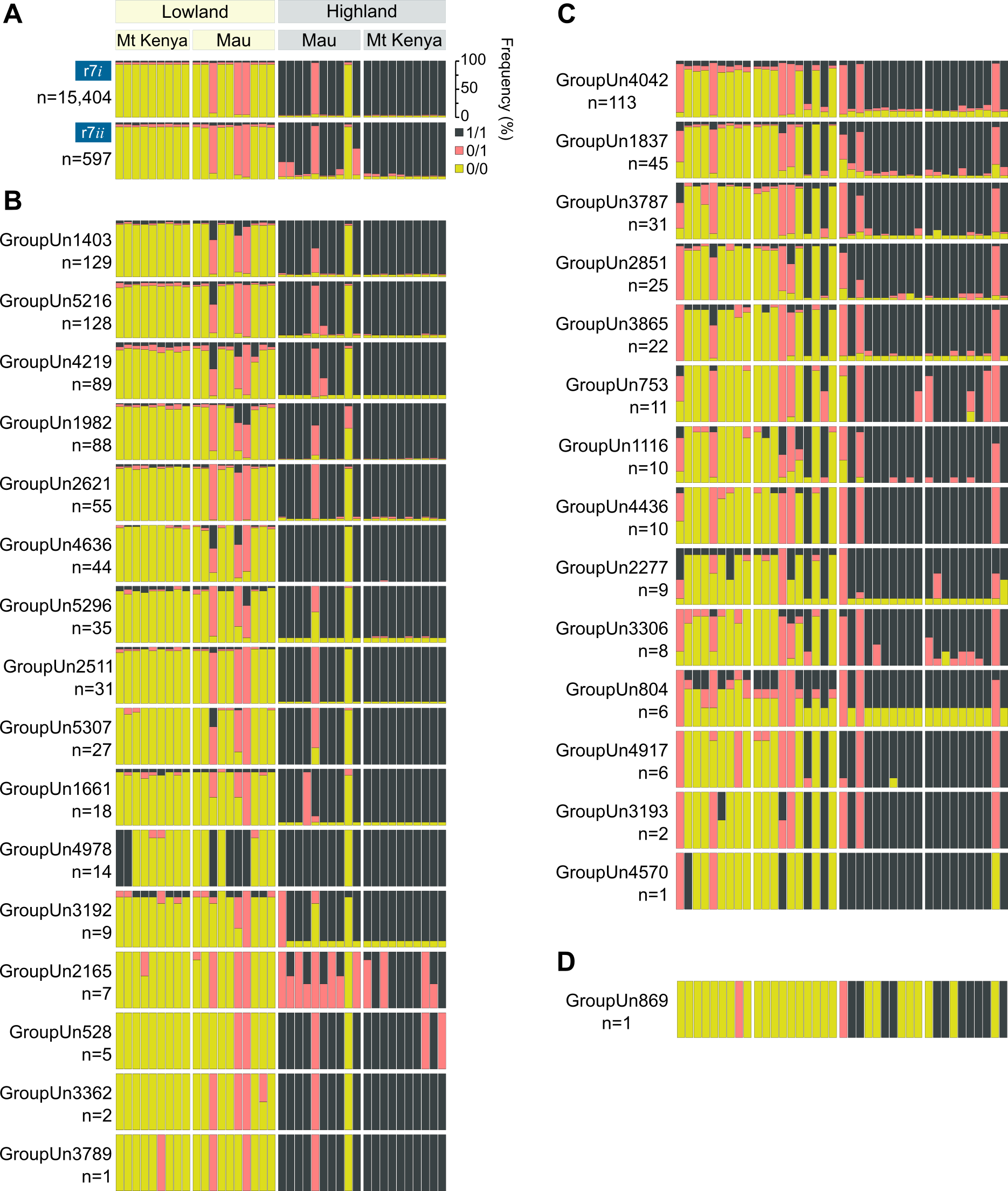

Supplement: S3 Fig — (A) Genotype and haplotype distributions on r7i and r7ii at SNPs that diverge between highland and lowland populations. At every genotype, a sample can be homozygous for the reference allele (0/0), homozygous for the non-reference allele (1/1) or heterozygous (0/1). Bar plots indicate the proportions of each genotype for all divergent SNPs (FST>0.5). Sample order as in Fig 4. (B) 16 unplaced scaffolds have similar genotype and haplotype distribution to r7. Symbols as in (A). (C) 14 scaffolds have similar genotype and haplotype distribution to r9. Symbols as in (A). (D) One scaffold without similarity to either r7 or r9. Symbols as in (A). (TIF) [file pgen.1006792.s003.tif]

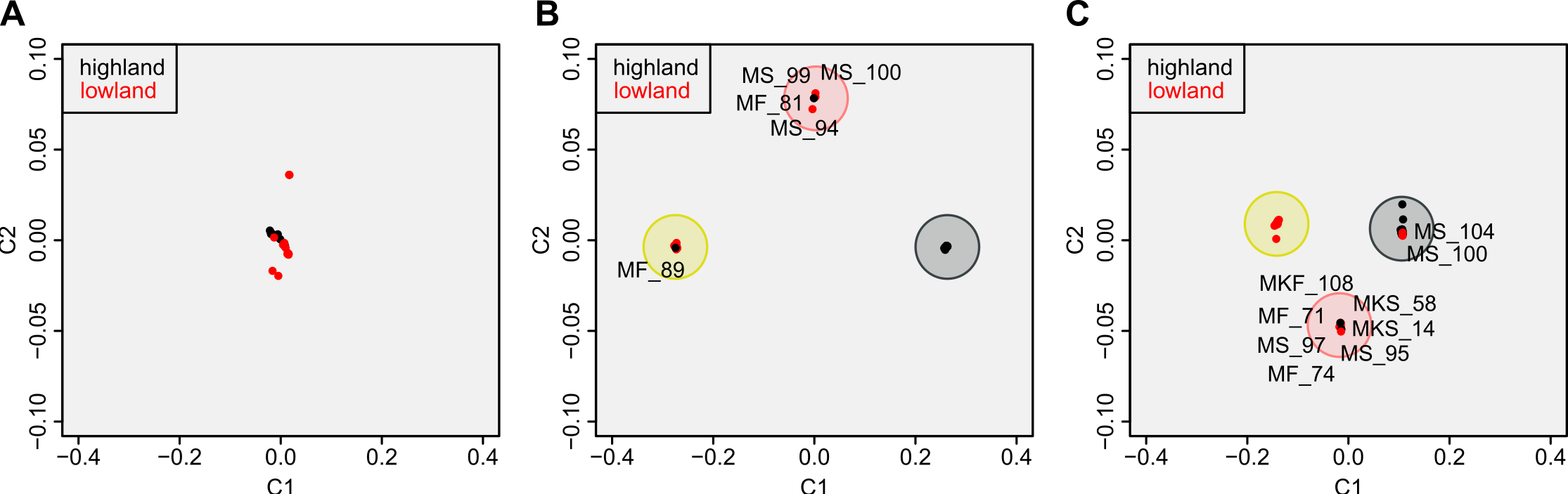

Supplement: S4 Fig — (A) Clustering based on all SNPs outside of the r7 and r9 regions. (B) Clustering in the r7 region on chromosome 7. Yellow circle indicates samples that are homozygous for the lowland haplotype. Grey circle indicates samples that are homozygous for the highland haplotype. Pink circle indicates samples that are heterozygous. Heterozygous samples and outlier samples with the opposite haplotype compared to the expected are labeled. (C) Clustering in the r9 region on chromosome 9. Symbols as in B. (TIF) [file pgen.1006792.s004.tif]

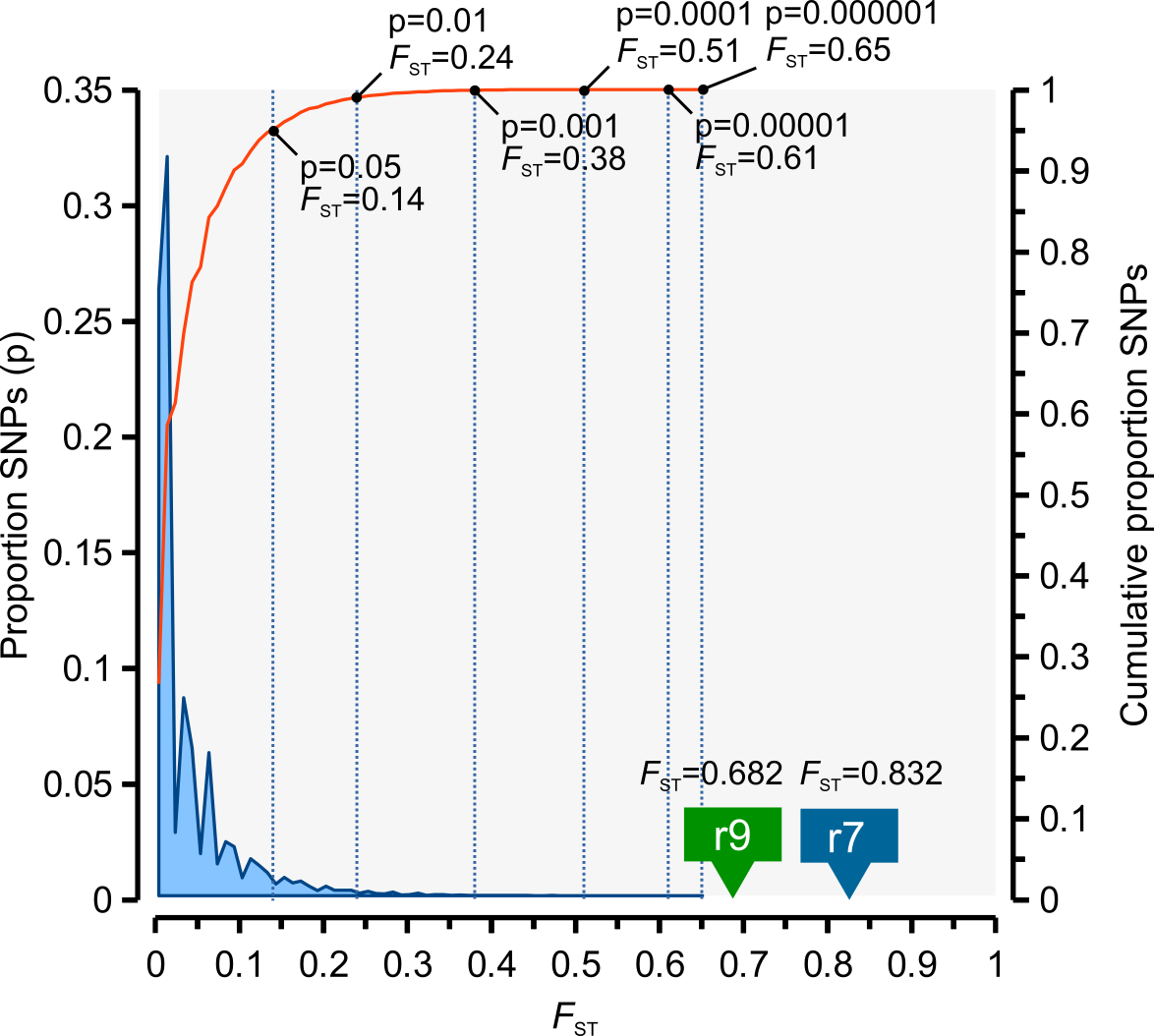

Supplement: S5 Fig — 1 million simulated SNPs were binned according to 0.01 intervals. Blue area represents SNP FST distribution (y1-axis). Red line is the cumulate proportion of SNPs (y2-axis). Black dots indicate the proportion (p) of SNPs above an FST threshold (p = 0.05 is the top 50,000 SNPs; p = 0.01 top 10,000 SNPs; p = 0.001 top 1,000 SNPs; p = 0.0001 top 100 SNPs; p = 0.00001 top 10 SNPs; p = 0.000001 top 1 SNP). Blue and green markers indicate the respective haplotype frequency differences at r7 and r9. (TIF) [file pgen.1006792.s005.tif]

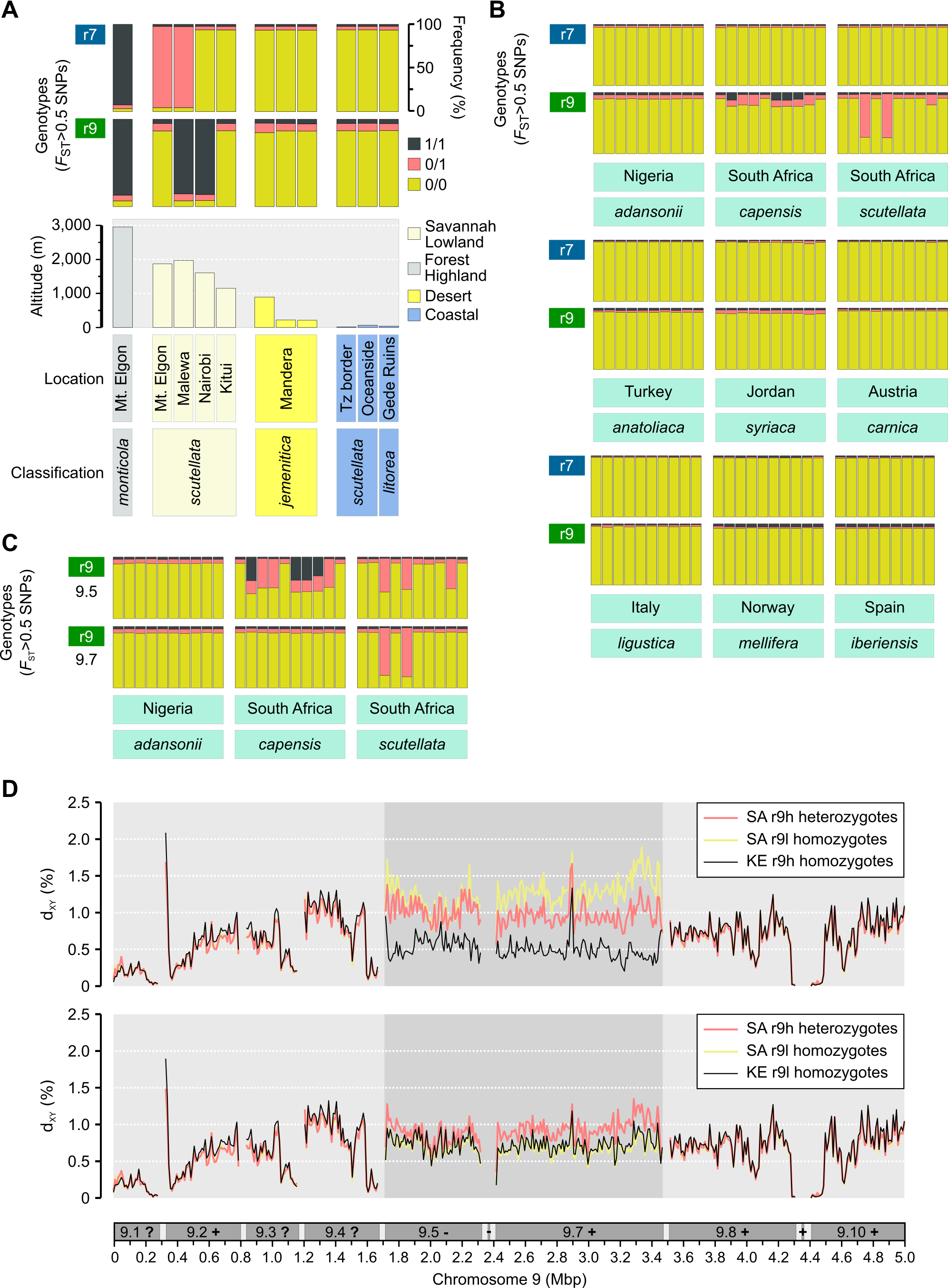

Supplement: S6 Fig — (A) Haplotypes detected for the 11 samples sequenced by Fuller et al. Color codes and bottom panels as in Fig 4. Sample order as in S1B Table. (B) A global sample of honey bees from [25]. Symbols as in Fig 4. Sample order as in S1C Table. (C) The r9 region for the African samples and subdivided for the two main scaffolds (scaffold 9.5; n = 2,231 SNPs; scaffold 9.7; n = 6,208 SNPs). (D) Divergence (dXY) between the two South African (SA) scutellata samples that appear to be heterozygous for the r9h highland haplotype (scu_3 and scu_5) and the Kenyan (KE) bees that are either homozygous for r9h (upper plot) or r9l (lower plot). dXY between South African (SA) scutellata samples homozygous for the lowland haplotype and the same Kenyan bees indicated in yellow. dXY within either group of Kenyan bees indicated in black. (TIF) [file pgen.1006792.s006.tif]

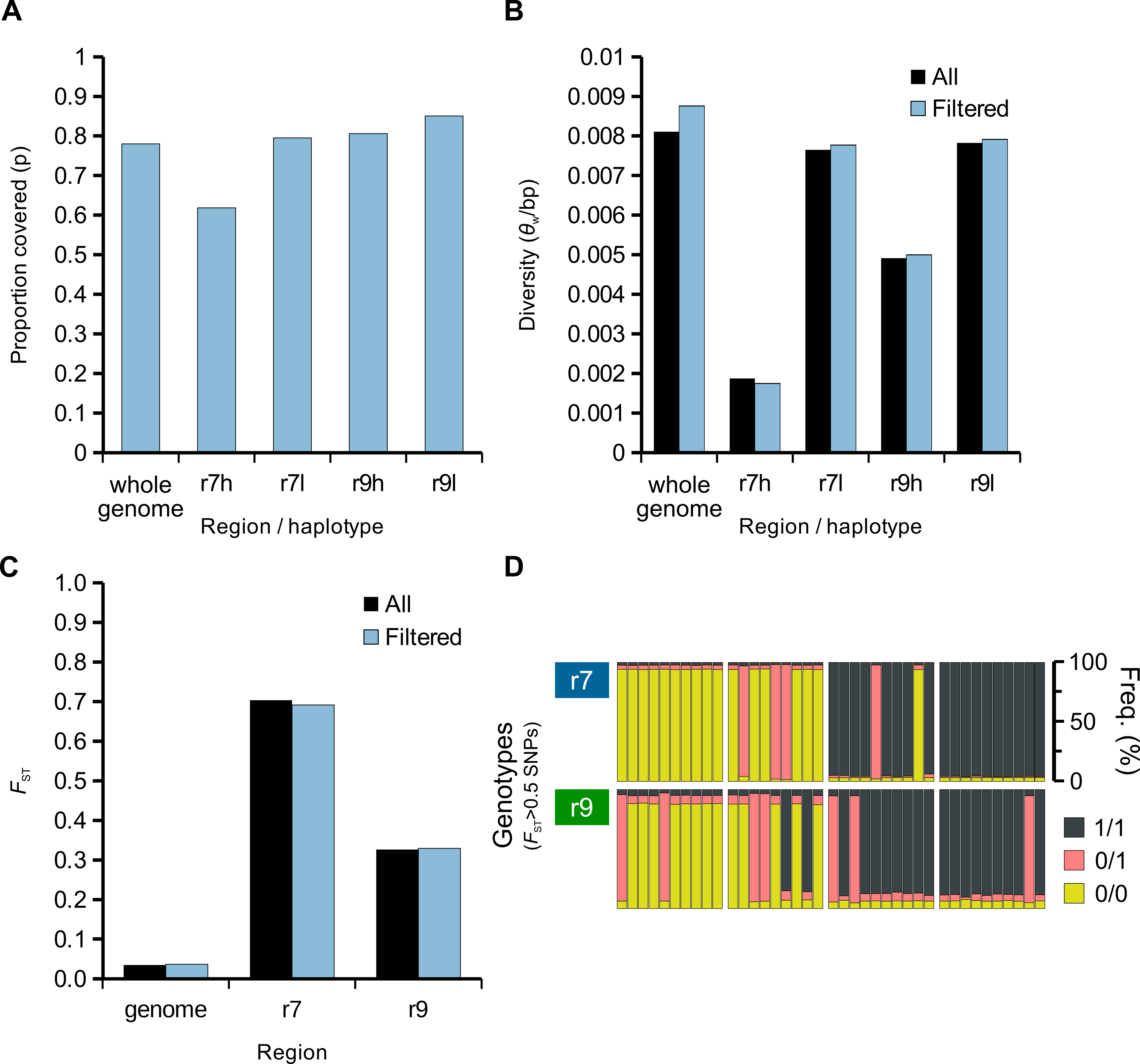

Supplement: S7 Fig — (A) Proportion of retained sites across the genome or divergent regions r7 and r9 after stringent filtering for mapping depth and sample coverage (see Results section for filters; r7h = r7 highland haplogroup; r7l = r7 lowland haplogroup; r9h = r9 highland haplogroup; r9l = r9 lowland haplogroup). (B) Average genetic diversity across the regions in A based on all data or after filtering. (C) FST between highland and lowland bees based on all data or after filtering. (D) Haplotype patterns for r7 and r9 after filtering (as compared to Fig 4). (TIF) [file pgen.1006792.s007.tif]

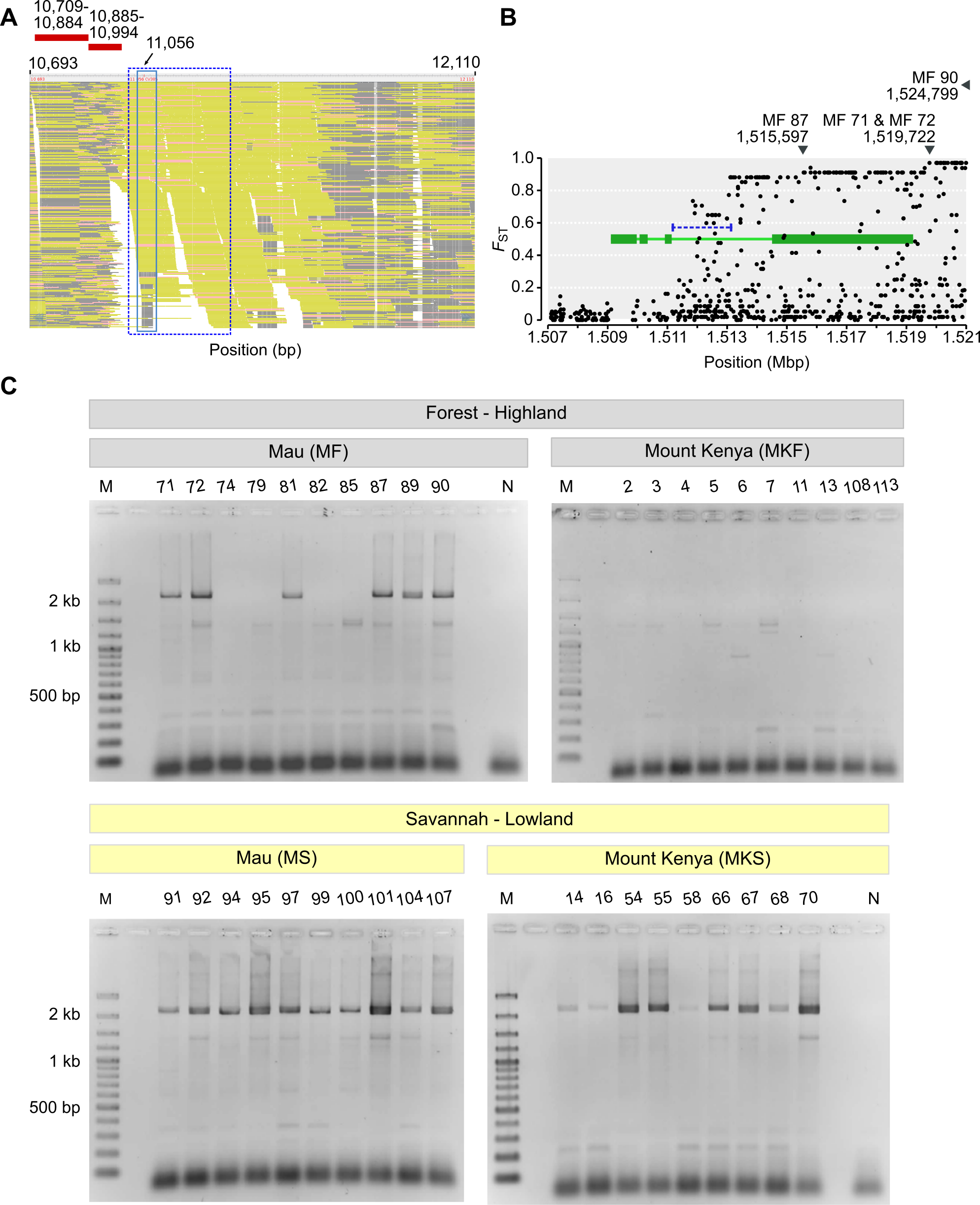

Supplement: S8 Fig — (A) Tablet visualization of read mapping across the putative breakpoint at the start of the r7 haplotype using. Reads from bees with r7 lowland haplotypes are yellow. Reads from bees with r7 highland haplotypes are grey. Reads from heterozygous samples are pink. Light blue box indicates spuriously mapped region in highland samples and contains the first outlier SNP at position 11,056 bp. Dashed blue box indicates a potential breakpoint region where no full read pairs from highland samples map. Red bands indicate location of AluI-like elements detected with BLAST. (B) SNPs across the octopamine receptor gene AmOctβ2R (GB49696). Gene body depicted in green (thin lines = introns; thick lines = exons). Dashed blue line indicates a putative breakpoint and amplification target in intron 3. Triangles indicate points where four Mau (MF) samples switch from being heterozygous for highland haplotypes to being homozygous. (C) Gel pictures of PCR products after attempting to amplify across the region indicated in (B). (TIF) [file pgen.1006792.s008.tif]

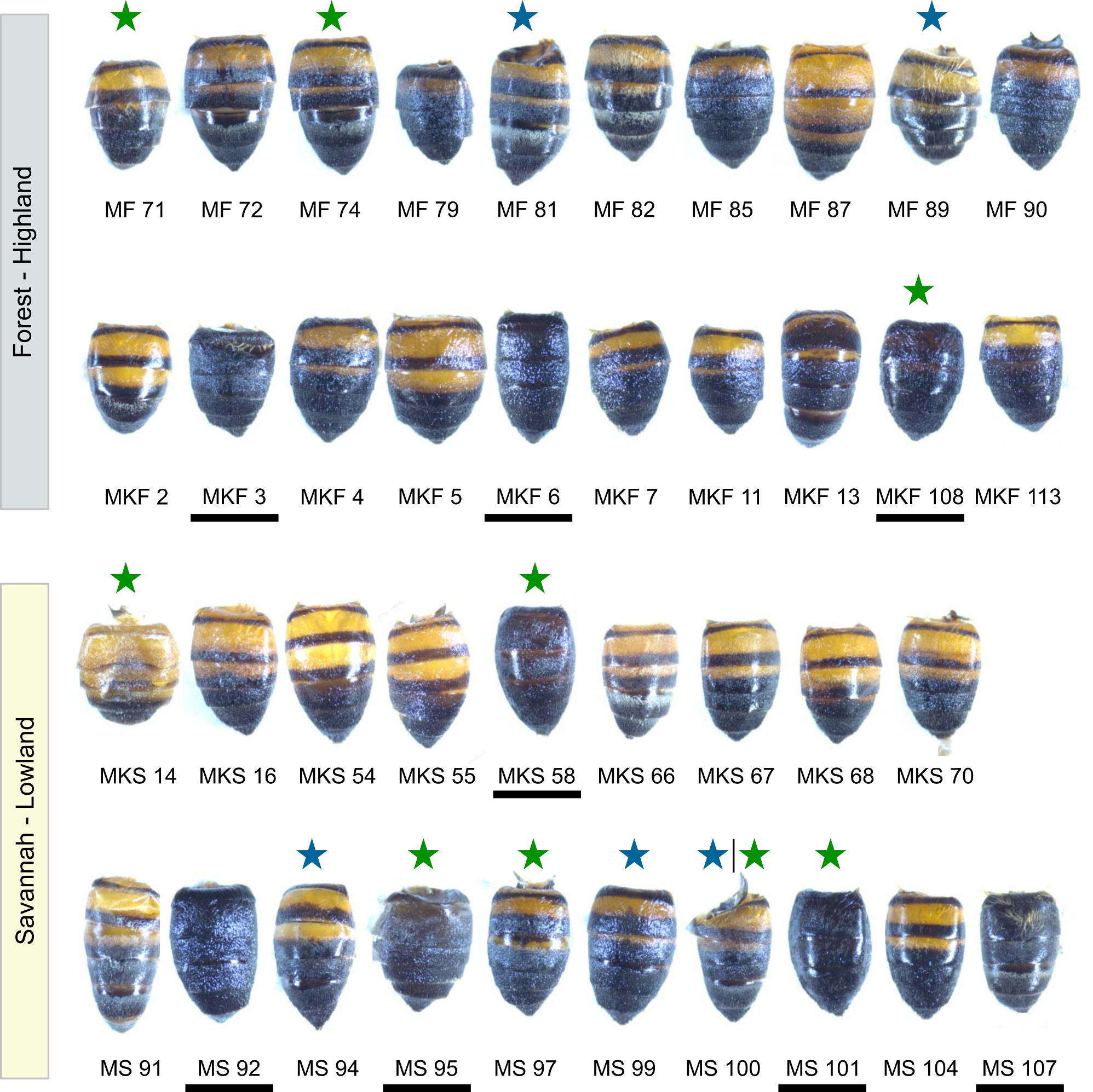

Supplement: S9 Fig — Stars indicate samples with contrasting haplotypes for r7 (blue) or r9 (green) from the common haplotype in either habitat (see Fig 4). Black bars indicate individuals with back/dark pigmentation across all tergites of the abdomen. (TIF) [file pgen.1006792.s009.tif]
